# Differential Effects of a First-in-Class IKK_α_ Inhibitor on Trabecular and Cortical Bone in a Human Prostate Cancer Xenograft Model

**DOI:** 10.1007/s00223-026-01558-y

**Published:** 2026-07-25

**Authors:** Abdullah Al-Jeffery, Feier Zeng, Antonia Sophocleous, Silvia Marino, Marco Ponzetti, Mattia Capulli, Andrew Paul, Robin Plevin, Ann C. M. Fowles, Simon P. Mackay, Nadia Rucci, Aymen I. Idris

**Affiliations:** 1 Department of Oncology and Metabolism, Medical School, Beech Hill Road, Sheffield, S10 2RX England, UK; 2 Bone and Cancer Group, Edinburgh Cancer Research Centre, Western General Hospital, University of Edinburgh, Crewe Road, Edinburgh, EH4 2XR Scotland, UK; 3 Department of Life Sciences, European University Cyprus, Nicosia, Cyprus; 4 Department of Biotechnological and Applied Clinical Sciences, University of L’Aquila, L’Aquila, Italy; 5 Strathclyde Institute of Pharmacy and Biomedical Sciences, University of Strathclyde, 161 Cathedral Street, Glasgow, G4 0NR Scotland, UK

**Keywords:** IKK_α_, NFkB, Prostate cancer, Bone metastasis, Osteolysis

## Abstract

IKK_α_, a key component of canonical and non-canonical NFκB signalling, is implicated in prostate cancer and bone metabolism. Here, we validated the anti-proliferative effects of selected molecules from a series of first-in-class IKK_α_ inhibitors against a panel of human prostate cancer models and then showed that the potent anti-tumour and highly IKK_α_-selective SU^1349^ reduced the ability of human PC3 cells to migrate and invade in vitro, induce osteolysis ex vivo, and cause trabecular bone loss in mice. Paradoxically, mice treated with SU^1349^ exhibited cortical bone loss. Histomorphometrical in vivo and functional in vitro studies confirmed that SU^1349^ suppressed osteoclastogenesis, but both enhanced and reduced osteoblast number and activity. Mechanistically, despite SU^1349^ being an inhibitor with a significant selectivity for IKK_α_ over IKK_β_, it inhibited both canonical and non-canonical NFκB signalling in PC3 cells, osteoclasts, and osteoblasts, and reduced the levels of several PC3-secreted, NFκB-regulated factors. This study suggests that IKK_α_ inhibitors could be of therapeutic value in the treatment of prostate cancer-induced osteolysis. However, osteoblast inhibition may limit their usefulness as osteoprotective agents.

**Graphical Abstract:**

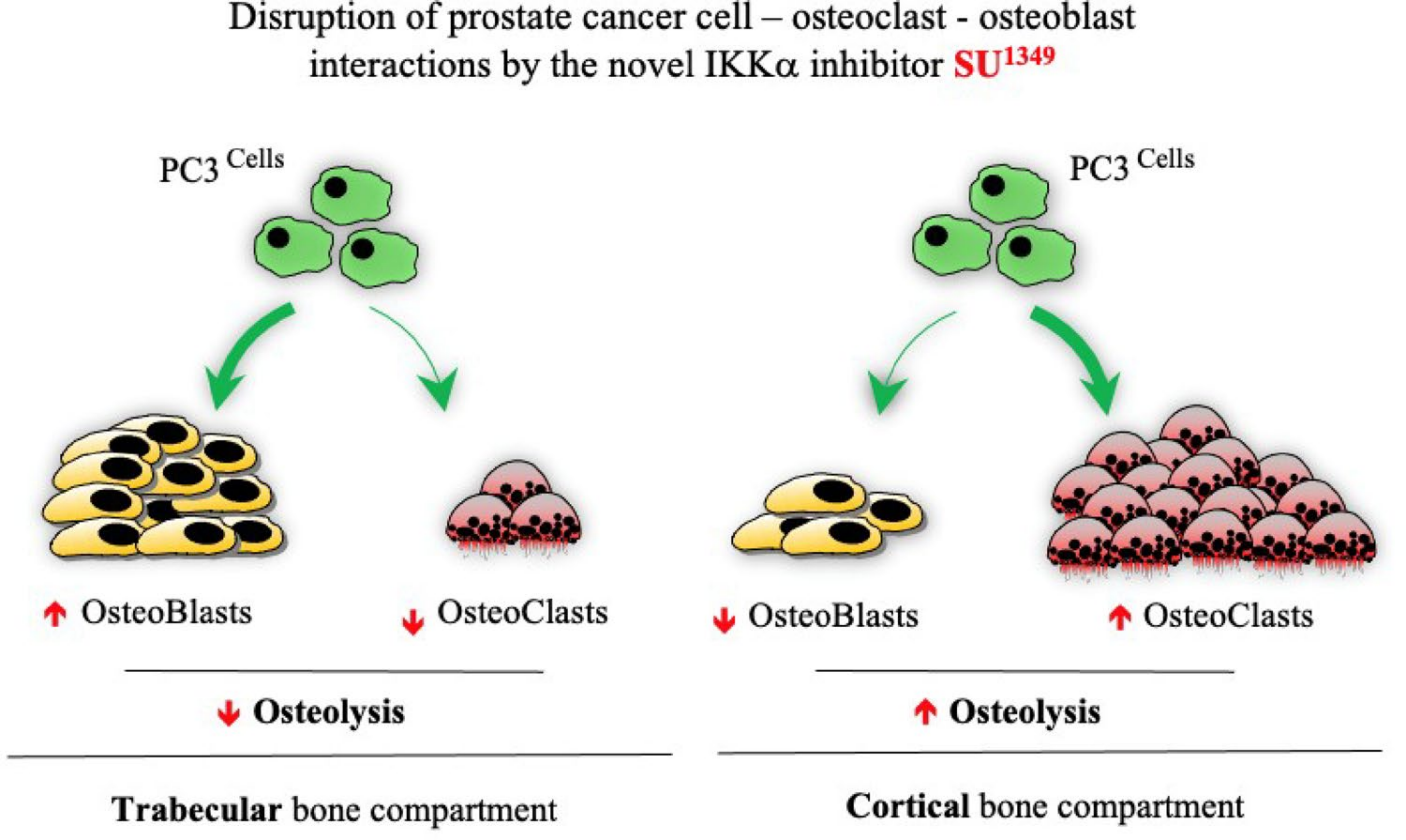

**Supplementary Information:**

The online version contains supplementary material available at 10.1007/s00223-026-01558-y.

## Introduction

IKK_α_ and IKKβ play key but divergent roles in the regulation of global NF-κB signalling and many aspects of cellular transcription [1, 2]. IKKβ regulates the activation of the canonical NF-kB pathway via activation of p65 RelA-p50 heterodimers [3–5] leading to the generation of multiple pro-inflammatory mediators in a variety of cell types which can support disease progression. Whilst IKK_α_ has been shown to have a lesser role in the canonical NF-kB pathway [2, 5], it is pivotal in the activation of the non-canonical NF-kB pathway, catalysing the phosphorylation and proteolytic processing of p100 NF-κB2, which in turn liberates distinct NF-κB dimers, typically p52/RelB complexes, and initiates transcription of a specific subset of genes. As IKK_α_ and IKK_β_ have specific cellular functions [1, 6–8], the selective inhibition of one isoform over the other could represent divergent approaches to new therapeutic interventions in inflammatory-based diseases and cancer.

The IKK_α_/NFkB axis is dysregulated in prostate cancer [9–12], and IKKα has been shown to regulate the survival of castration-independent prostate cancer cells under inflammatory conditions [13]. Using the TRAMP mouse model [14], Luo and colleagues showed that IKK_α_ expression correlates with initiation and growth of prostatic tumours, and implicated active nuclear IKK_α_ in the metastatic phase of the disease [9, 15]. Studies by several other workers further consolidated the association of IKK_α_ expression and activity with advanced prostate cancer in animal models and poor survival rate in patients [15–17].

Prostate cancer cells preferentially metastasise to the skeleton [18–21]. Skeletal-related events, particularly bone pain and fractures, are serious complications associated with secondary prostate cancer in bone [20, 22]. Bone metastases of prostatic origin are predominately osteosclerotic, and predominately associated with an increase in osteoblast number and activity [21, 23]. Prostate cancer cells in bone (aka osteotropic cells) cooperate with osteoblasts in enhancing the formation, survival and resorptive capabilities of multi-nucleated osteoclasts in the tumour microenvironment, thereby orchestrating the development of osteolytic lesions [23–25]. A myriad of tumour-, matrix- and bone cell-derived factors implicated in prostate cancer—bone cell crosstalk also regulate IKK_α_ activity [23, 25–29]. Various osteolytic factors implicated in prostate cancer metastasis, such as receptor activator of NFκB ligand (RANKL), tumour necrosis factor alpha (TNFα) and interleukin-1 beta (IL-1β), trigger the activation of canonical IKK_α_/IKK_β_/IKK_g_ signalling, which involves the phosphorylation of IKK_α_, IKK_β_, and IkB that in turn free p65/p50 dimers to translocate to the nucleus [30–33]. Activation of non-canonical IKK_α_/NFkB signalling by cluster of differentiation 40 ligand (CD40L) and Lipopolysaccharide (LPS) leads to p100 phosphorylation and the subsequent nuclear translocation of RelB [30, 31, 34, 35]. Several genetic studies in mice have demonstrated that deficiency in IKK_α_ or RelB reduces osteoclast number and enhances bone volume [36–38]. Whilst these studies clearly implicate IKK_α_ in prostate cancer as well as bone remodelling, the osteoprotective effect of IKKα inhibition in models of prostate cancer associated bone disease has not been investigated. These efforts have been hampered, at least in part, by the lack of commercially available agents that selectively inhibit the kinase activity of IKK_α_ [39, 40].

In 2017, Anthony et al. described the discovery of a series of selective IKKα inhibitors, but with modest potency in cells [41]. This series has since been superseded by a second generation of IKK_α_ inhibitors with improved physicochemical properties and cellular potency that represent primary selective and potent pharmacological tools that can be used to interrogate the signalling function of IKK_α_ [42]. Using a combination of in vitro, ex vivo and in vivo models of prostate cancer cell—osteoclast—osteoblast interaction, we validated the anti-proliferative effects of selected members of this series against a panel of human castration-independent cell-lines with different degrees of metastatic, bone-seeking and osteotropic properties, to show that the potent anti-tumour and highly selective IKK_α_ inhibitor SU^1349^ reduced the ability of human PC3 cells to influence bone cell activity and to cause osteolysis in mouse calvarial ex vivo and the trabecular compartment of bone in adult mice. Of therapeutic importance, mice treated with SU^1349^ exhibited bone loss at the cortical compartment. Thus, IKK_α_ inhibitors show promise in the treatment of secondary prostate cancer in bone. However, cortical bone loss may limit their therapeutic usefulness.

## Materials and Methods

### IKKα Inhibitors

The selective IKK_α_ kinase inhibitors SU^1261^ and SU^1349^ were synthesised, purified and characterised as described by Mackay and co-workers in [42]. IKK_α_ inhibitors were dissolved in dimethyl sulfoxide (DMSO) and stored at − 20 °C. All in vitro and ex vivo cultures were treated with vehicle (0.1% (v/v) DMSO) or an IKK_α_ inhibitor at the stated concentration in the presence or absence of human PC3 cells or their derived factors (20% (v/v)), for the desired period. In vivo, mice received intraperitoneal (IP) injection of vehicle (1% (v/v) DMSO in phosphate buffered saline, PBS) or SU^1349^ (20 mg/kg/thrice-weekly).

### Cell Lines and Culture Condition

The human hormone-sensitive LNCaP cells and their metastatic C4-2 and osteotropic C4-2B4 clones, human castration-independent PC3 prostate adenocarcinoma, human Saos-2 and mouse MC3T3 osteoblast-like, and mouse RAW 264.7 macrophage-like cell lines were purchased from ATCC (Manassas, VA, USA), and were cultured in a standard D-MEM, unless stated otherwise. Tissue culture medium (D-MEM and alpha-MEM) and fetal bovine serum (FBS) were purchased from ThermoFisher (Leicestershire, UK). Standard DMEM and alpha-MEM tissue culture mediums were supplemented with 10% (v/v) fetal calf serum (FCS), glutamine (2mM), penicillin (100U/ml), and streptomycin (100 µg/ml) (Sigma-Aldrich, Dorset, UK).

### Generation of Conditioned Medium

For studies involving prostate cancer-derived factors, human PC3 cells were cultured to 80% confluence, and then incubated in serum free medium for an additional 16 h, conditioned medium (CM) was removed, filtered (0.22 μm filter diameter) and added to cultures at 20% (v/v), for the desired period [43]. Level of PC3-derived factors in conditioned medium was determined by Proteome Profiler Human XL Cytokine Array Kit (ARY022, R&D Systems, Abingdon, UK), according to the manufacturer’s instructions.

### Generation of Stable Cell Lines

The expression of IKK_α_ was genetically manipulated in human PC3 cells using a lentiviral delivery system. Briefly, IKK_α_ over-expression or GFP vector DNA (5 µl) was used to transform bacteria (One Shot TOP10^®^ E. coli system, Invitrogen, Paisley, UK), as per manufacturer’s instructions. Construct DNA was recovered, quantified using NanoDrop TM and stored at − 20 °C. IKK_α_-shRNA expressing lentivirus was generated by transfecting HEK293ET cells (5 µg of vector DNA, 5 µg of gag and pol DNA, 5 µg of pMD2.G vector, 40 µl of polyethylenimine (PEI) transfection reagent and 450 µl of standard DMEM medium). The shRNA vectors (TRC human individual clones: TRCN-504 and − 505) and the pLKO control used to generate stable knockdowns of IKK_α_ expression in human PC3 cells were obtained from ThermoFisher (Leicestershire, UK). IKK_α_ over-expression and GFP control constructs (pMXs vectors with a Puromycin selection cassette) were a gift from Professor Yousef Abu-Amer (Washington University School of Medicine, USA). Successfully transfected human PC3 cells were selected with puromycin (1 µg/ml), maintained in a selection medium for at least two passages, and the efficiency of knockdown/over-expression was validated by Western blot analysis (supplementary Fig. S1) [44].

### Assessment of In Vitro Osteoblastogenesis

Osteoblast viability, differentiation and bone nodule formation were assessed in cultures of primary calvarial osteoblasts and human Saos2 osteoblast-like cells [45]. For assessment of osteoblast differentiation, cells were cultured in standard alpha-MEM (8 × 10^3^ cells/well) and differentiation was assessed by measuring alkaline phosphatase (Alk Phos) activity as previously described [45]. For bone nodule assays, human Saos-2 cells (1.5 × 10^5^ cells/well) were cultured in osteogenic medium (standard alpha-MEM supplemented with L-ascorbic acid (50 ng/ml) and β-glycerophosphate (2.0 mM), Sigma-Aldrich, Dorset, UK) for up to 21 days. Bone nodule formation was evaluated by alizarin red (ALZ) staining [45]. Osteoblast number was assessed in all cultures by AlamarBlue assay [45].

### Assessment of In Vitro Osteoclastogenesis

Osteoclast formation was studied using calvarial—bone marrow cell co-culture, and RANKL and M-CSF stimulated human and mouse osteoclast cultures [46]. Human osteoclasts were generated from peripheral blood mononuclear CD14^+^ monocytes isolated from healthy volunteers by positive bead selection (Miltenyi Biotec) and Ficoll-Pacque separation [46]. Briefly, CD14^+^ monocytes were cultured in MCSF (100ng/ml) for 48 h and mature osteoclasts were generated following the addition of RANKL (100ng/ml) and MCSF (25ng/ml) [46]. In osteoclast cultures where mouse RAW 264.7 pre-osteoclasts were used, only RANKL was added. In mouse osteoblasts—bone-marrow cell co-cultures, calvarial osteoblasts isolated from the calvarial bone of 2-day old mice and were plated into 96-well plates (8 × 10^3^ cells/well), and bone marrow (BM) cells (2 × 10^5^ cells/well) isolated from mouse long bones were added [46]. Co-cultures were maintained in standard alpha-MEM supplemented with 1,25-(OH)2-vitamin D3 (10 nM) for up to 7 days and IL1β (100 ng/ml) was added in the last three days [43]. Mouse macrophage colony stimulating factor (M-CSF) was obtained from R&D Systems (Abingdon, UK) and human 1,25-(OH)2-vitamin D3 and IL1β were obtained from (Sigma-Aldrich, Dorset, UK). Human receptor activator of NFkB ligand (RANKL) was a gift from Patrick Mollat (Galapagos SASU, France) [47]. Mature osteoclasts (3 or more nuclei) and their precursors were identified using Tartrate-resistant Acid Phosphatase (TRAcP) staining [46].

### Assessment of In Vitro Cell Motility

The 2D directed and 3D random migration of human PC3 cells were assessed using time-lapse microscopy and T-scratch analysis program [48]. Cell velocity and accumulated distance travelled by cells were measured using the Chemotaxis and Migration tool in ImageJ [48]. The transwell assay was used to measure the invasive ability of human PC3 cells [48]. Briefly, RANKL (100ng/ml) was used as the chemoattractant, transwell inserts (8 μm, Corning, UK) containing fixed, invasive cells were stained with haemotoxylin and eosin, and the number of invasive cells were quantified using ImageJ [48].

### Western Blotting

Western blot analysis was used to detect the expression of total and phosphorylated canonical IkB and non-canonical P100 in human PC3 cells, osteoblasts, and osteoclasts and, their precursors cultured in standard media, as previously described [44]. Briefly, cells were incubated in serum free tissue culture medium for 16 h prior the addition of vehicle or an IKK_α_ inhibitor prepared in serum free media. Total protein (50–100 µg) was resolved by SDS-PAGE, transferred onto PVDF membranes (BioRAD, UK) and immunoblotted with appropriate antibodies, using horseradish peroxidase-conjugated secondary antibody (Jackson labs, UK), according to manufacturer’s instructions. Rabbit anti-actin was obtained from Sigma-Aldrich (Dorset, UK) and other primary rabbit antibodies IKK_α_, p-p100, p100, p52, pIkB, and IkB were purchased from Cell Signalling Biotechnology (London, UK). Membranes were visualised using chemiluminescence (Amersham, UK) on a ChemiDocMP imaging system (Biorad, Exeter, UK) [44].

### Animal Experiments

All in vivo and ex vivo protocols that involves animals and their tissue were approved by the Ethics Committee at the Universities of Edinburgh (Scotland, United Kingdom) and L’Aquila (Italy) and were conducted in accordance with the United Kingdom Home Office regulations.

### Mouse Calvarial Model of Osteolysis

The effects of selective IKK_α_ inhibitor SU^1349^ on ex vivo osteolysis associated with human PC3 cells was studied using the mouse calvarial organ culture system [43]. Briefly, mouse calvarial bones were isolated from 2-day-old CD1 mouse neonates (Harlan Laboratories, UK), each half was placed on stainless steel rafts, and cultured in standard alpha-MEM medium in the presence of human PC3 cells (10^3^ cells/well). Cultures terminated after 7 days, and bone volume (BV) was measured by micro-computed tomography (microCT, resolution, 5 μm) [49].

### Intracardiac Injection of Human PC3 Prostate Cancer Cells

The effect of the selective IKK_α_ inhibitor SU^1349^ on prostate cancer-induced bone disease was studied following intra-cardiac injection of human PC3 cells in BALB/c nu/nu athymic mice (Charles River, Milan, Italy). Briefly, 4-week-old male BALB/c nu/nu athymic mice were anesthetised with intraperitoneal injections (IP) of pentobarbital (60 mg/kg), and intra-cardiacly inoculated with luciferase-expressing human PC3 cells (1 × 10^5^ cells, 0.1 ml of PBS) into the left ventricle. Animals were divided into two groups (8 mice/group) and received intraperitoneal injection of 150 µl solution of SU^1349^ dissolved in mannitol (20 mg/kg/thrice/weekly) or vehicle control. Dosage and treatment regimens (20 mg/kg/thrice-weekly) were based on the in vivo pharmacokinetic profiling parameters reported for SU^1349^ [42], which demonstrate adequate plasma exposure above the cellular IC50 for IKK_α_ inhibition [42]. Animals were monitored daily for cachexia (evaluated by reduction in body weight), behaviour and survival. The development of metastasis was monitored by bioluminescence imaging and X-ray analysis (36 KPV, 10 s) using a Cabinet X-ray system (Faxitron model n.43855 A; Faxitron X‐ Ray Corp., IL, USA) and IVIS Spectrum CT In Vivo Imaging System (PerkinElmer, UK). Radiographs were scanned using Bio‐Rad scanning densitometer (Hercules, CA, USA).

### Micro-Computed Tomography

Assessments of trabecular and cortical bone volume and architecture were performed using ex vivo micro-computed tomography (microCT) [49]. Briefly, mouse tibia and calvarial bones bone were scanned using a Skyscan 1172 instrument (Brucker, Belgium) set at 60 kV and 150µA and at a resolution of 19 μm (long bone) and 5 μm (calvarial bone). Images were reconstructed using Skyscan NRecon and CTAn softwares (Brucker, Belgium). Measurement of trabecular bone was carried out at the proximal tibial metaphysis (500 frames distal to the growth plate), whereas cortical bone measurement was carried out at the proximal diaphysis (100 frames situated 700 frames distal to the reference line).

### Bone Histomorphometry

Assessment of in vivo osteoclast and osteoblast number and activity in mouse tibia was performed using static and dynamic histomorphometry [50]. Briefly, excised mouse bones were fixed, underwent standard stages of dehydration, decalcified and embedded in paraffin wax and sectioned (4 μm, Leica microtome, Solms, Germany), according to standard techniques [50]. Randomly selected cross sections were stained with TRAcP and counter stained with haematoxylin [50]. Bone histomorphometry was performed on trabecular and cortical bone (3 sections, 20 μm apart, per sample) at 200µM distal from the growth plate using Osteomeasure image analysis system (Osteometrics, USA). Human tissue microarrays from Prostate cancer patients (PR242b, US Biomaz Inc., FisherScientific, UK) were dehydrated, blocked with goat serum (5% (v/v)) and incubated in mouse anti-IKK_α_ monoclonal-antibody (5 mg/ml, Novus Biologicals, Bio-Techne Ltd., UK) and a secondary SS Boost (Cell Signalling Biotechnology, London, UK). Haematoxilin was used as a counterstain.

### Statistical Analysis

Statistical analysis was carried using GraphPad Prism 7 for Windows (GraphPad Prism, La Jolla California, USA). Results were reported as mean ± standard deviation (SD) unless otherwise stated. Student’s* T* test was performed to determine the significance level of differences between two sets of results. For multiple comparison between groups analysis of variance (ANOVA) followed by a post-hoc test. A* p*-value of 0.05 or below was considered statistically significant. The half maximal inhibitory concentration (IC_50_) was calculated by non-linear regression analysis using the 4 parameter slope fit equation for dose-inhibition response.

## Results

### Anti-Proliferative Effect of the IKK_α_ Inhibitor SU^1349^ In Vitro

To date, and to our knowledge, no inhibitor with significant selectivity for IKK_α_ over IKKβ has been tested for anti-tumour and osteoprotective effects in models of secondary prostate cancer in bone. Thus, we first performed a concentration-response investigation that examined a number of selective IKK_α_ inhibitors, including SU^1261^ (kinase activity (Ki): 0.01 µM for IKK_α_ and 0.6 µM for IKK_β_) and it’s highly IKK_α_-selective analogue SU^1349^ (Ki: 0.01 µM for IKK_α_ and 3.35 µM for IKK_β_) [42]. Initially, we examined the in vitro proliferation of a panel of human prostate cancer cell-lines to establish whether these effects correlated with their cell-free kinase inhibitory activity against IKK_α_ and IKK_β_ previously determined using a dissociation enhanced ligand fluorescent immunoassay (DELFIA) (Table 1). The cell lines used include clones of the human castration-independent PC3 and hormone-dependent LNCaP cells that exhibit different degrees of metastatic, bone-seeking and osteotropic properties. As shown in Table 1, SU^1261^ and SU^1349^ reduced the viability of LNCaP cells and their metastatic C4-2 and osteotropic C4-2B4 clones, and PC3 cells and their osteotropic clone PC3-BT in a concentration-dependent manner. The concentration of SU^1349^ that half-maximally inhibited cell viability (IC_50_) was lower than SU^1261^ (Table 1). Consistently, SU^1349^ exhibited a higher potency against and selectivity for IKK_α_, when compared to IKK_β_—evident by the concentrations needed to selectively inhibit IKK_α_ kinase activity (Table 1). Notably, the IC_50_ values for cellular viability across these prostate cancer cell lines also correlated with the inhibitory concentrations recently demonstrated by SU^1349^ against cellular pharmacodynamic markers of IKK_α_ activity (LTα_1_β_2_-stimulated IKKα-mediated p100 phosphorylation and p52/RelB nuclear translocation) in a PC3 cell line (IC_50_ = 0.2 µM and 0.15 µM, respectively) [42]. The non-active control, SU^1257^, had no significant effect on prostate cancer cell proliferation at concentrations up to 30.0µM (Table 1).

**Table 1:** Effects of the first-in-class SU family of agents on prostate cancer cell proliferation in vitro

Agent	Human prostate cancer cell viability (IC50, µM)				
	LNCaP	C4-2	C4-2B4	PC3	PC3-BT
SU^1257^	> 30	> 30	> 30	> 30	> 30
SU^1261^	0.69 ± 0.01	0.39 ± 0.02	0.48 ± 0.02	1.46 ± 0.28	0.95 ± 0.11
SU^1349^	0.25 ± 0.05	0.16 ± 0.01	0.24 ± 0.03	1.35 ± 0.36	0.24 ± 0.01
SU^1361^	0.85 ± 0.09	0.43 ± 0.03	0.61 ± 0.10	1.94 ± 0.7	0.75 ± 0.04
SU^1411^	1.04 ± 0.14	1.33 ± 0.78	1.21 ± 0.63	1.24 ± 0.09	1.37 ± 0.13
SU^1266^	2.81 ± 0.19	3.28 ± 0.87	2.72 ± 0.37	2.21 ± 0.12	1.88 ± 0.1
SU^1087^	11.0 ± 0.01	14.22 ± 3.92	10.12 ± 2.36	10.4 ± 0.01	19.07 ± 6.82

Growth assays and calculation of half maximal inhibitory concentrations (IC50) have been performed as described under “Materials and Methods”. Values are expressed as means ± sd and are obtained from 3 independent experiments. NT denotes not tested

### Anti-Migratory and Anti-Invasive Effects of the IKK_α_ Inhibitor SU^1349^ In Vitro

**Fig. 1:**
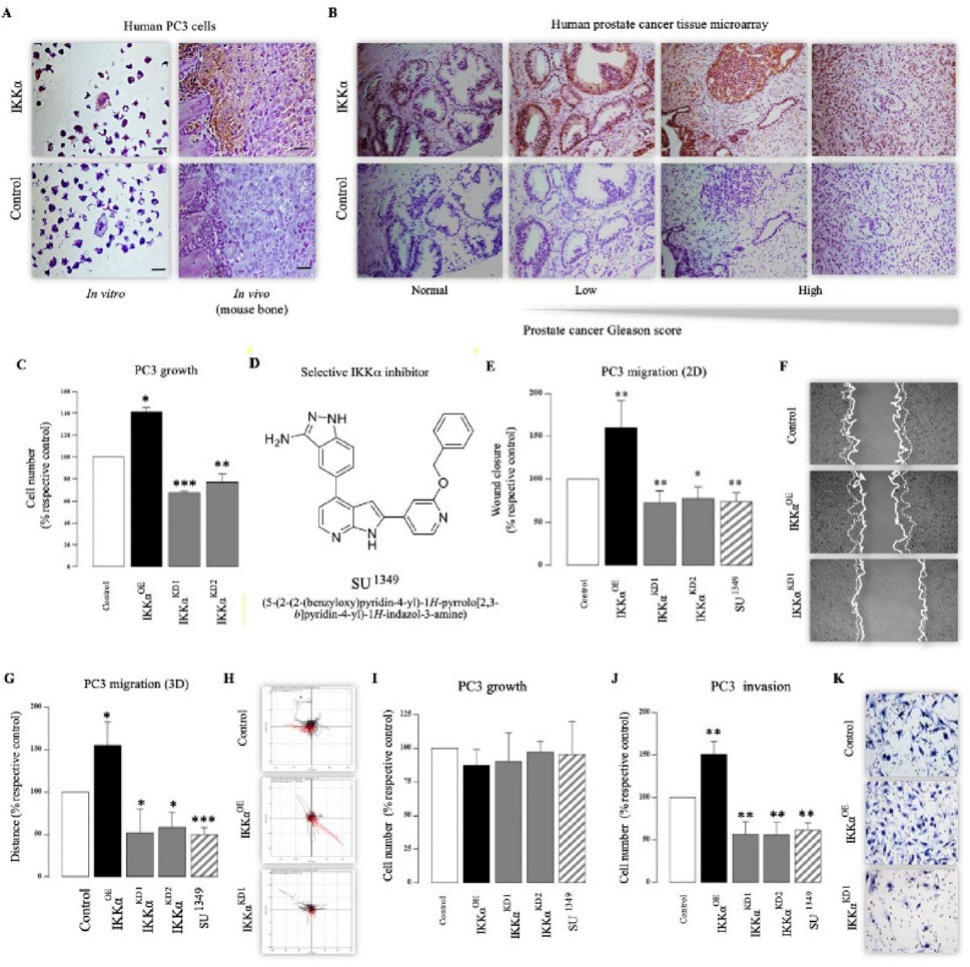
IKK_α_ inhibition reduces the metastatic behaviour of human PC3 cells. A. In vitro and in vivo expression of IKK_α_ (brown) in human PC3 prostate cancer cells in culture (cell plugs, left panel) and in tibial bone marrow of BALB/c mice following intra-cardiac injection (right panel). **B** In situ immunohistochemical expression of IKK_α_ (brown) in patients with different degree of prostate cancer metastasis and Gleason score (low, 3, to high, 5). Control (lower panels) denotes Immunoglobulin G (IgG) stained samples. **C** In vitro growth of human PC3 cells overexpressing (IKK_α_^OE^) and deficient in (IKK_α_^KD1^ and ^KD2^) IKKα and their mock control cultured in serum-enriched complete media (FCS, 20% v/v) after 72 h. Cell growth assessed by AlamarBlue assay. **D** The chemical structure of the selective IKKα inhibitor SU^1349^. **E**, **F** In vitro 2D-directed migration of human PC3 cells overexpressing (IKKα^OE^) and deficient in (IKK_α_^KD1^ and ^KD2^) IKK_α_ and mock control cells treated with vehicle (0.1% DMSO) or the selective IKK_α_ inhibitor SU^1349^ (1.0µM) for 12 h. Cultures were maintained in FCS-free media and assessed for 2D-directed cell movement by wound healing assay. Representative photomicrographs from the experiment described are shown in panel (**F**). **G**, **H** In vitro 3D-random migration of the human PC3 cells described in the experiments in panels B-C after 12 h, as assessed by ImageJ. Summary plots showing directionality and distance of tracked human PC3 cells random migration from the experiment described are shown in panel (**H**). Red and black lines represent up or down end points for tracked cells. **I** In vitro growth of the human PC3 cells from the experiment described in panels **E–H** after 16 h, as assessed by AlamarBlue assay. **J**, **K** In vitro invasion of human PC3 cells overexpressing (IKK_α_^OE^) and deficient in (IKK_α_^KD1^ and ^KD2^) IKK_α_ and mock control cells treated with vehicle (0.1% DMSO) or the selective IKK_α_ inhibitor SU^1349^ (0.3µM) for 72 h, as assessed by Trans-well invasion assay. Representative photomicrographs from the experiment described are shown in panel (**K)**. Values in graphs are mean (expressed as % of vehicle control) ± standard deviation (s.d.) and are obtained from 3 independent experiments. * *p* < 0.05; ** *p* < 0.01; *** *p* < 0.001 from vehicle or mock control

Previous studies confirmed that constitutive IKK_α_ activity is higher in human castration-independent prostate cancer cells when compared to hormone-dependent and primary prostatic cells [13]. Here, we show that IKK_α_ is constitutively expressed in the castration-independent PC3 cells in vitro (Fig. 1A, left) and in the long bones of BALB/c mice following intra-cardiac injection (Fig. 1, right). IKK_α_ expression in PC3 cells in bone is comparable to that observed in tissue microarray from patients with high prostate cancer Gleason score (Fig. 1A, B). Encouraged by this finding, we went on to stably knockdown and over-express IKK_α_ in human PC3 cells (supplementary Fig. S1), and to demonstrate that PC3 cells deficient in IKK_α_ exhibited significant reduction in growth when cultured in serum rich medium for 72 h, whereas IKK_α_ over-expression was stimulatory (Fig. 1C). Next, we examined the effects of the over-expression as well as pharmacological (using SU^1349^, Fig. 1D) and genetic inhibition of IKK_α_ in the in vitro metastatic behaviour of the human PC3 cells. These experiments confirmed that IKK_α_ over-expression significantly enhanced the directed 2D (Fig. 1E, F) and random 3D (Fig. 1G, H) migration of human PC3 cells when cultured in serum-free medium for 12 h, and demonstrated that these effects were completely abolished by IKK_α_ knockdown or exposure to the selective IKK_α_ inhibitor SU^1349^ (1.0µM) (Fig. 1D). It is important to note that neither genetic manipulation of IKK_α_ nor SU^1349^ treatment (1.0µM) affected PC3 cell viability under the conditions described (Fig. 1I). This excludes the possibility that the anti-migratory effects observed under these conditions (Fig. 1C–H) were mediated by cytotoxicity. Furthermore, IKK_α_ knockdown and treatment with SU^1349^ (0.3µM) reduced the invasive capabilities of human PC3 cells, whereas IKK_α_ over-expression was stimulatory (Fig. 1J and K).

### SU^1349^ Reduces the Osteolytic Behaviour of Human PC3 Cells In Vitro and Ex Vivo

**Fig. 2:**
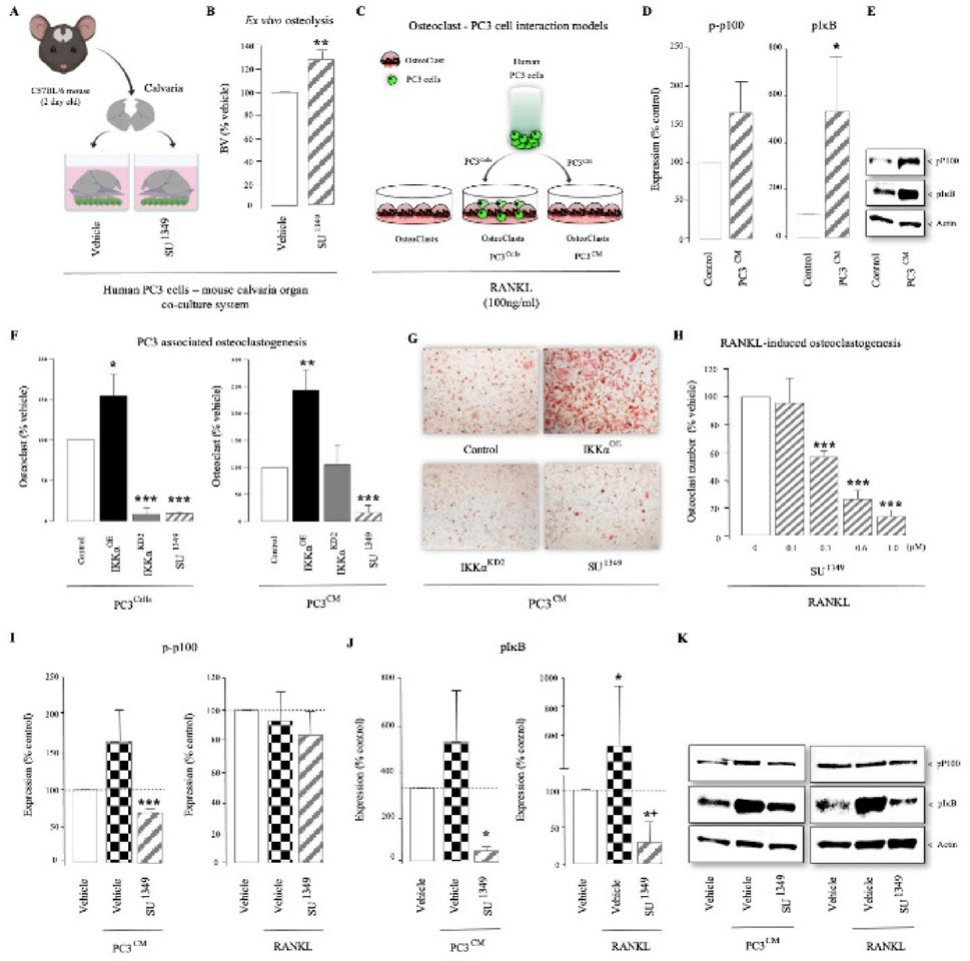
SU^1349^ reduces the osteolytic behaviour of human PC3 cells.** A** Graphic representation of the ex vivo mouse calvarial organ—PC3 cell co-culture system used to study the effects of vehicle (0.1% DMSO) and the selective IKK_α_ inhibitor SU^1349^ (1.0µM) on PC3-associated osteolysis after 5 days. Schematic was created with BioRender.com. **B** Ex vivo bone volume (BV) of the mouse calvaria from the experiment described in panel A, as assessed by microCT scanning. **C** Graphic representation of in vitro models used to study used the effects of IKK_α_ inhibition on the interactions between osteoclasts and PC3 prostate cancer cells and their derived factors (conditioned medium, CM, 20% v/v) in RANKL-stimulated mouse bone marrow (left) and RAW.267 pre-osteoclast-like macrophages (middle and right). **D**, **E** Western blot quantification of the phosphorylation of non-canonical p100 (left), canonical IkB (right) and actin expression in mature osteoclasts in cultures of RANKL-generated RAW.267 cells exposed to conditioned medium (CM, 20% v/v) from human PC3 cells for 16 h in serum free medium. Representative photomicrographs of Western blots from the experiment described in panel (**E**). **F**, **G** In vitro osteoclast formation in cultures of RANKL-stimulated mouse RAW.267 cells co-cultured with (left) or exposed to conditioned medium (CM, 20% v/v) from (right) human PC3 cells overexpressing (IKKα^OE^) and deficient in (IKK_α_^KD1^ and ^KD2^) IKK_α_ and their mock control treated with vehicle or SU^1349^ (1.0 µM) for 72 h. Representative photomicrographs of TRAcP-stained mature osteoclasts and their precursors from the experiment described are shown in panel (**G**). **H** In vitro osteoclast formation in cultures of RANKL (25ng/ml)-induced RAW.267 cells treated with vehicle or SU^1349^ (0–1.0 µM) for 72 h. Multi-nucleated osteoclasts (3 nuclei or more) and their precursors were visualized by TRAcP staining assay. Values in graphs are mean (expressed as % of vehicle control) ± standard deviation (s.d.) and are obtained from 3 independent experiments. * *p* < 0.05; ** *p* < 0.01; *** *p* < 0.001 from vehicle or mock control

**Fig. 3:**
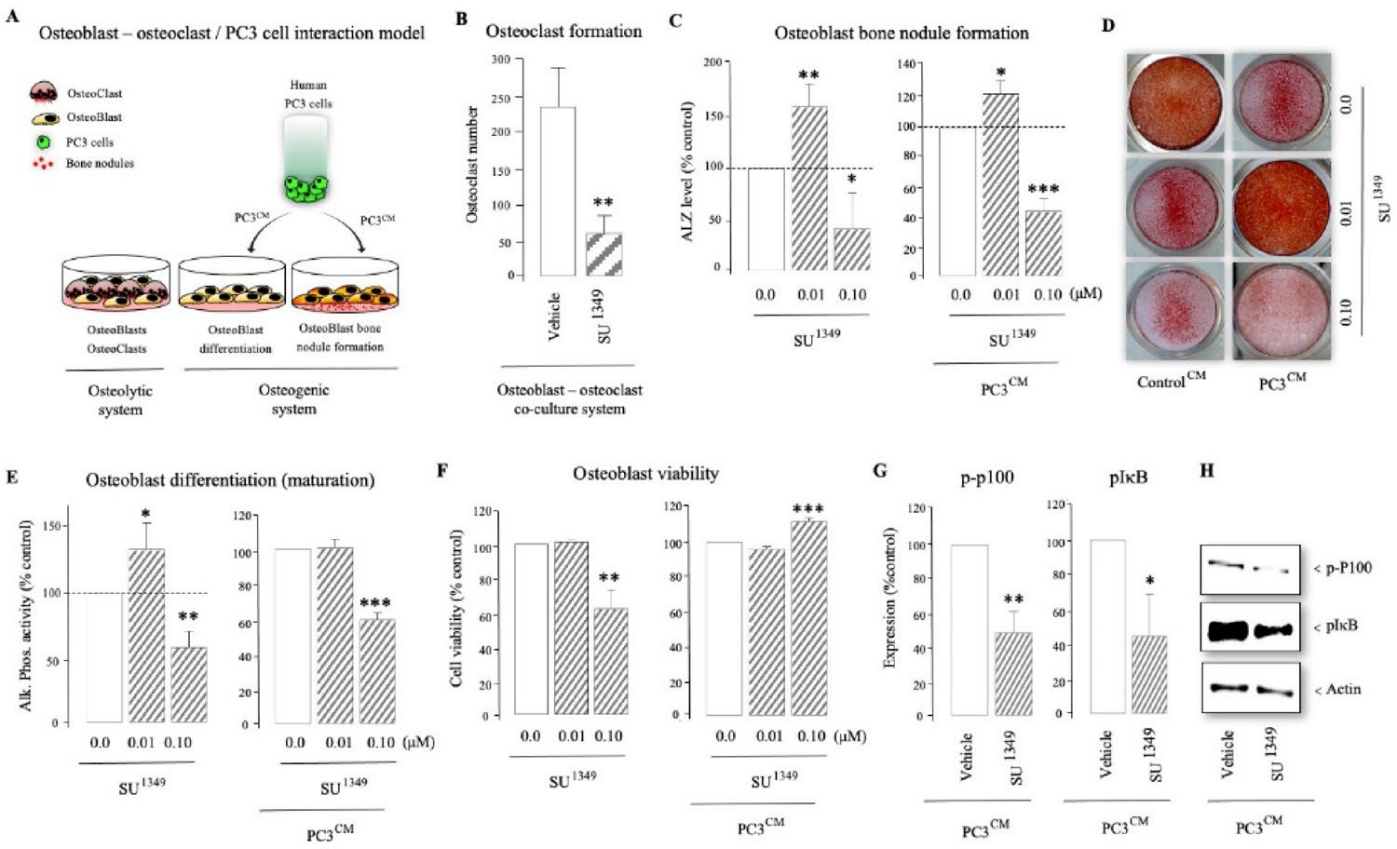
SU^1349^ enhances and reduces PC3-associated osteoblastogenesis.** A** Graphic representation of in vitro models used to study the effects of IKK_α_ inhibition on the interactions between osteoblasts, osteoclasts and PC3 prostate cancer cells and their derived factors (conditioned medium, CM, 20% v/v) under osteolytic and osteogenic conditions. **B** In vitro osteoclast formation in cultures of mouse calvarial osteoblasts and bone marrow macrophages treated with vehicle (0.1% DMSO) or the selective IKK_α_ inhibitor SU^1349^ (1.0 µM) for 96 h, as assessed by TRAcP staining. **C** In vitro bone nodule formation in cultures of human Saos-2 pre-osteoblasts treated with vehicle or SU^1349^ at the indicated concentration in the absence (left) and presence (right) of conditioned medium (CM, 20% v/v) from human PC3 cells after 21 days, as assessed by Alazarin Red (ALZ) assay. Representative photomicrographs of ALZ-stained bone nodule formation from the experiment described are shown in panel (**D**). **E** In vitro differentiation and maturation of human Saos-2 pre-osteoblasts from the experiment described in panel C, as assessed by alkaline phosphatase (Alk. Phos.) assay. **F** In vitro growth of human Saos-2 pre-osteoblasts from the experiment described in panels (**C**–**E**), as assessed by AlamarBlue assay. **G**, **H** Quantification of the phosphorylation of non-canonical p100 (left) and canonical IkB (middle), and total actin expression in human Saos-2 pre-osteoblasts treated with vehicle or SU^1349^ (0.1µM) for 6 h. Cultures were maintained in serum-free media and protein expression was assessed by Western blot. Representative photomicrographs of Western blots from the experiment described are shown in panel (**H**). Values in graphs are mean (expressed as % of vehicle control) ± standard deviation (s.d.) and are obtained from 3 independent experiments. Dotted line denotes control. * *p* < 0.05; ** *p* < 0.01; *** *p* < 0.001 from vehicle or mock control

Human PC3 cells metastasise to the skeleton (Fig. 1A) [51], however the effects of pharmacological inhibition of IKK_α_ on prostate cancer-induced bone cell activity and remodelling have not been investigated. Thus, we validated the osteoprotective effects of SU^1349^ ex vivo using the mouse calvarial organ—human PC3 cell system (Fig. 2A). As shown in Fig. 2B, treatment with SU^1349^ (1.0µM) significantly enhanced bone volume in the mouse calvarial co-cultured with human PC3 cells for 7 days. To examine the anti-osteolytic properties of SU^1349^, we utilized a number of in vitro models of PC3 cell—osteoclast—osteoblast interactions (Figs. 2C and 3A). First, we used Western blot analysis to confirm if PC3 derived factors (conditioned medium, CM) activate IKK-mediated non-canonical and canonical NFkB activation in osteoclasts. As shown in Fig. 2D and E, exposure of mature osteoclasts to conditioned medium (CM, 20% v/v) from human PC3 cells significantly enhanced the phosphorylation of IkB within the canonical NF-kB pathway, but with a non-significant trend towards increased p100 phosphorylation as a component of the non-canonical NF-kB cascade (*p* = 0.12). Next, we showed that osteoclast number is significantly enhanced in RANKL-generated mouse RAW.267 osteoclasts co-cultured with human PC3 over-expressing IKK_α_ (Fig. 2F, left) or their derived factors (CM, 20%, v/v, Fig. 2F, right). Conversely, exposure of mouse RAW.267 osteoclasts to human PC3 cells deficient in IKK_α_, their derived factors (CM, 20%, v/v) or SU^1349^ (1.0µM) were inhibitory (Fig. 2F and G). In further support of the anti-osteoclastic properties of this agent, we show that SU^1349^ inhibited RANKL-induced osteoclast formation in the absence of PC3 cells or their derived factors (Fig. 2H). To explore the mechanism(s) by which SU^1349^ exerts its anti-osteolytic (Fig. 2B) and anti-osteoclastic (Fig. 2F–H) effects, we pretreated mouse RAW.267 pre-osteoclasts with SU^1349^ (10.0µM) for 1-hour prior exposure to RANKL (100ng/ml) or conditioned medium from human PC3 cells (CM, 20%, v/v). As shown in Fig. 2 (panels I to K), conditioned medium from human PC3 cells (CM, 20%, v/v) induced the phosphorylation of p100 (Fig. 2I, left) and IkB (Fig. 2J, right) in pre-osteoclasts, and these effects were completely prevented by SU^1349^ (10.0 µM). In contrast, RANKL induced the phosphorylation IkB—but not p100—in pre-osteoclasts and IkB activation in these cells was completely inhibited by SU^1349^ treatment (Fig. 2I–K, right).

### SU^1349^ Reduces and Enhances Osteoblastic Behaviour of Human PC3 Cells In Vitro

The bone-forming osteoblasts regulate the osteolytic behaviour of prostate cancer cells through the secretion of several IKK_α_-activating factors, such as RANKL and IL1β and CD40L [32–35]. With this in mind, we tested the effect of SU^1349^ on the ability of osteoblasts to regulate osteoclastogenesis in the presence and absence of human PC3 cells and their derived factors (Fig. 3A). Using the mouse calvarial osteoblast—BM cell co-cultures, we demonstrated that pretreatment of osteoblasts with SU^1349^ (0.3 µM) prior to the addition of mouse BM cells and IL1β (100 ng/ml) significantly inhibited osteoclast formation, further confirming the anti-osteoclastic properties of this agent. Osteoblasts produce osteosclerotic legions in prostate cancer patients [21, 23], and here we first confirm that exposure of human Saos-2 pre-osteoblasts to conditioned medium (20% v/v) from human PC3 cells significantly enhanced their ability to form bone nodule (Fig. S2A), mature (Fig. S2B) and grow (Fig. S2C) after 21 days (supplementary Fig. S2). Treatment of human Saos-2 pre-osteoblasts with SU^1349^ both stimulated (0.01 µM) and inhibited (0.10 µM) their ability to form bone nodule in the absence and presence of human PC3 derived factors (CM, 20% v/v). Furthermore, SU^1349^ stimulated (0.01µM) and inhibited (0.10 µM) the differentiation and maturation of human Saos-2 pre-osteoblasts in the cultures described, evident by the levels of Alkaline Phosphatase (Alk Phos) activity (Fig. 3E). Of note, treatment of human Saos-2 pre-osteoblasts with 0.10 µM SU^1349^ inhibited cell viability, whereas 0.01 µM had no significant effect. Interestingly, we detected an increase in viability in cultures of human Saos-2 pre-osteoblasts that were cultured in the presence of conditioned medium from human PC3 cells (CM, 20% v/v), indicating that prostate cancer derived factors enhance osteoblast number in our model. We also confirmed in samples prepared from human osteoblasts under similar conditions, in the presence of conditioned medium from human PC3 cells, that SU^1349^ (0.10µM) significantly inhibited basal p100 phosphorylation and IkB phosphorylation (Fig. 3G–H). In contrast, SU^1349^ failed to affect p100 and IkB phosphorylation at lower concentrations and/or in the absence of condition medium (data not shown).

### SU^1349^ Inhibits Trabecular Bone Loss in Mice Bearing Human PC3 Cells

**Fig. 4:**
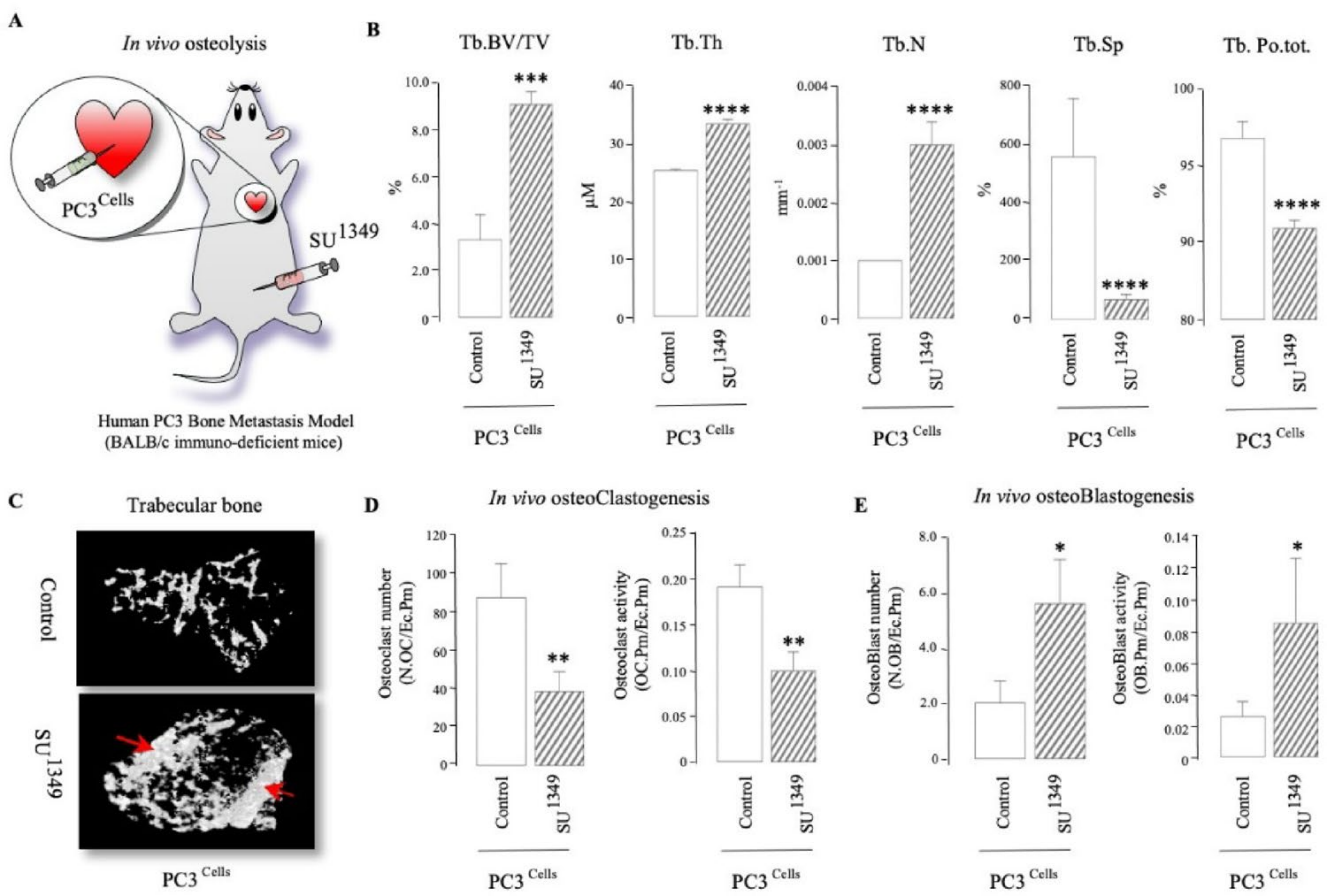
Administration of SU^1349^ reduces trabecular bone loss in mice bearing human PC3 cells.** A** Graphic representation of intra-cardiac injection of human PC3 cells into 8 weeks old adult BALB/c-nu/nu athymic mice treated with the selective IKK_α_ inhibitor SU^1349^ (*n* = 16, 20 mg/kg/thrice-weekly) or vehicle (*n* = 13) for 12 days. **B** In vivo trabecular bone volume (BV/TV, %), thickness (Tb.Th, µM), separation (Tb.Sp, %), number (Tb.N, mm^−1^) and total porosity (Tb.Po.tot, %) in the tibial metaphysis of the mice from the experiment described in panel (**A**). Bone parameters were assessed by microCT, and representative photomicrographs of microCT scans of the trabecular compartment at the tibial metaphysis of mice from the experiment described are shown in panel (**C**). Red arrows denotes the formation of tumour-induced ectopic bone. **D** In vivo histomorphometric analysis of osteoclast number (N.OC/Ec.Pm) and activity (OC.Pm/Ec.Pm) in the trabecular compartment at the tibial metaphysis of the mice from the experiment described in panels (**A**–**C**). **E** In vivo histomorphometric analysis of osteoblast number (N.OB/Ec.Pm) and activity (OB.Pm/Ec.Pm) in the tibial metaphysis of the mice from the experiment described in panels (**A**–**D**)

To validate the osteoprotective properties of SU^1349^ in vivo, we evaluated its effect on bone cell activity and bone volume and architecture in immuno-deficient BALB/c mice bearing human PC3 cells (Fig. 4A). Detailed microCT analysis of the metabolically active [52], trabecular bone at the tibial metaphysis showed that administration of SU^1349^ (*n* = 16, 20 mg/kg/thrice-weekly) significantly increased trabecular bone volume (Tb.BV/TV), trabecular thickness (Tb.Th), trabecular number (Tb.Th), and reduced trabecular separation (Tb.Sp) and porosity (Tb.Po.tot) (Fig. 4B and C) in mice, when compared to vehicle treated group (*n* = 13). Close examination of bone architecture in microCT reconstructed images suggests that mice treated with SU^1349^ showed signs of formation of tumour-induced ectopic bone formation (Fig. 4C, red arrows). Histomorphometric analysis of bone cell number and activity in histological sections showed that SU^1349^ treatment reduced osteoclast number (Fig. 4D, left) and resorptive activity (Fig. 4D, right), and enhanced osteoblast number (Fig. 4E, left) and activity (Fig. 4E, right). Together, these results indicate that SU^1349^ enhances trabecular bone volume by exerting an anti-osteolytic and pro-osteoblastic effects in the mouse model described. Of note, mice treated with SU^1349^ (*n* = 16, 20 mg/kg/thrice-weekly) exhibited a non-significant trend towards reduced overt metastasis 17 (*p* = 0.3) and 22 (*p* = 0.4) days post inoculation (supplementary Fig. S5), thereby excluding any anti-metastatic effects.

### SU^1349^ Causes Cortical Bone Loss in Mice Bearing Human PC3 Cells

**Fig. 5:**
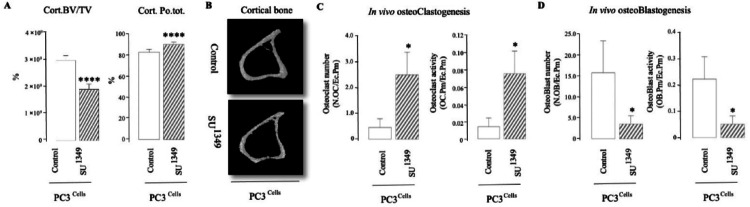
Administration of SU^1349^ enhances cortical bone loss in mice bearing human PC3 cells.** A** In vivo cortical bone volume (cort.BV/TV, %) and total porosity (Tb.Po.tot, %) in the tibial metaphysis of 8 weeks old adult BALB/c-nu/nu athymic mice following intra-cardiac injection of human PC3 cells and treatment with the selective IKK_α_ inhibitor SU^1349^ (*n* = 16, 20 mg/kg/thrice-weekly) or vehicle (*n* = 13) for 12 days. Bone parameters were assessed by microCT, and representative photomicrographs of microCT scans of the cortical compartment at the tibial metaphysis of mice from the experiment described are shown in panel (**B**). **C** In vivo histomorphometric analysis of osteoclast number (N.OC/Ec.Pm) and activity (OC.Pm/Ec.Pm) in the cortical compartment at the tibial metaphysis of the mice from the experiment described in panels (**A, B**). **D** In vivo histomorphometric analysis of osteoblast number (N.OB/Ec.Pm) and activity (OB.Pm/Ec.Pm) in the cortical compartment at the tibial metaphysis of the mice from the experiment described in panels (**A–C**)

Detailed microCT analysis of the relatively inert, cortical bone of mice from the experiment described in Fig. 4A revealed that administration of SU^1349^ (*n* = 16, 20 mg/kg/thrice-weekly) caused significant reduction in cortical bone volume (Cort.BV/TV) and enhanced bone porosity (Cort.Po.tot) (Fig. 5A, B). Histomorphometric analysis of cortical bone confirmed that SU^1349^ treatment enhanced osteoclast number (Fig. 5C, left) and activity (Fig. 5C, right), and reduced osteoblast number (Fig. 5D, left) and activity (Fig. 5D, right). Collectively, these findings suggest that administration of the selective IKK_α_ inhibitor SU^1349^ causes differential effect on trabecular and cortical bone in the mouse model of human prostate cancer described.

### SU^1349^ Disrupts Human PC3–Bone Cell Crosstalk

**Fig. 6:**
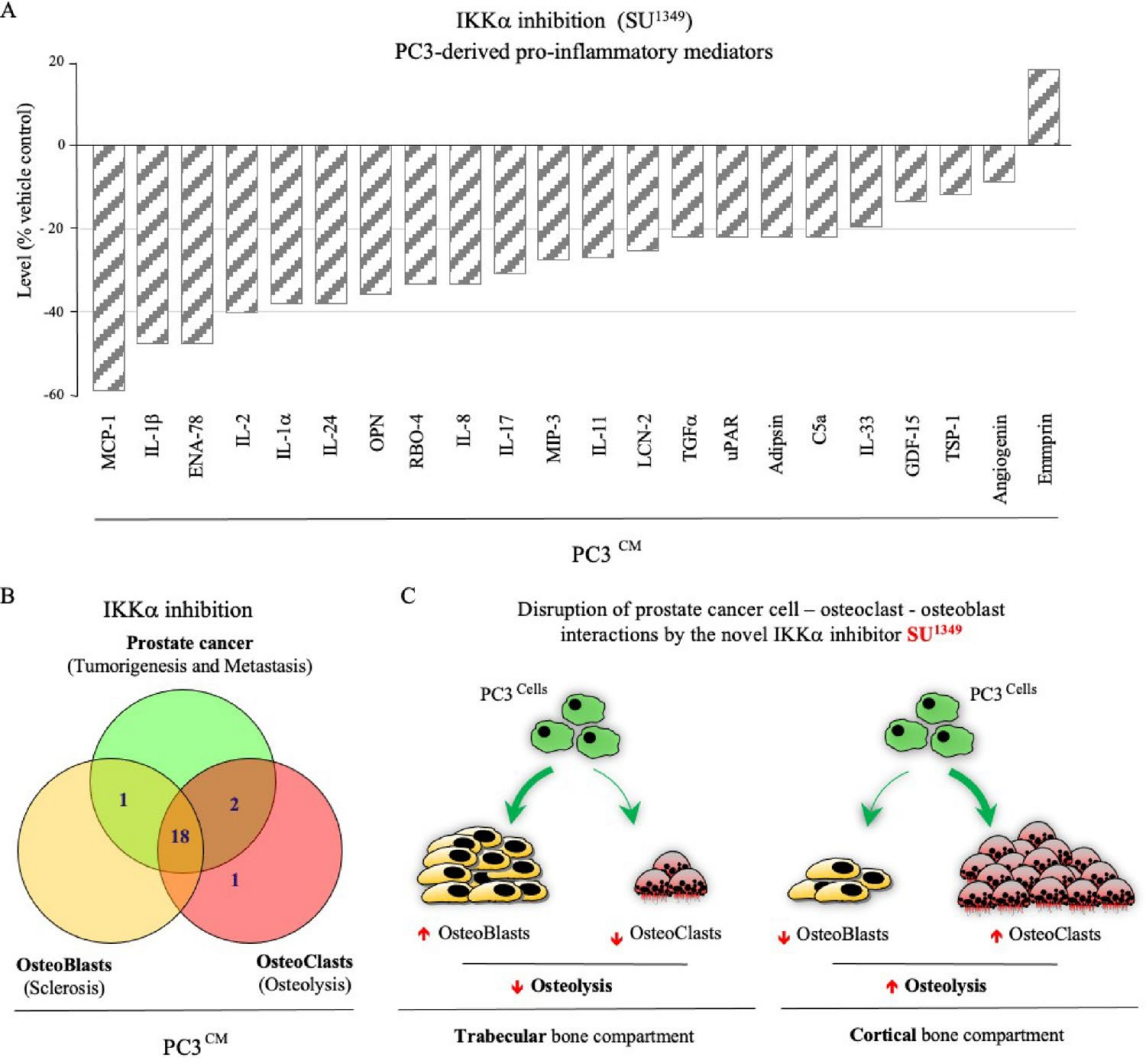
SU ^1349^ reduces the ability of human PC3 prostate cancer cells to secrete pro-inflammatory mediators in vitro. **A** Catalogue of differentially expressed inflammatory mediators in conditioned medium from human PC3 cells treated with vehicle or SU^1349^ (0.1µM) for 6 h. **B**. Venn-diagram of differentially expressed osteolytic, sclerotic and prostate cancer mediators in conditioned medium from the experiment described in panel (**A**). **C** A proposed model for disruption of prostate cell—osteoclast—osteoblast interactions by the IKK_α_ inhibitor SU^1349^ at the trabecular and cortical compartments of bone. The proposed model—when combined with previous studies in models of prostate cancer [9, 13], 15– [17] and bone metabolism [36–38]—suggests that IKK_α_ inhibitors such as SU1349 may be of value in the treatment of prostate cancer-induced bone disease. However, inhibition of osteoblastogenesis combined with cortical bone loss may limit their therapeutic usefulness

We employed an analytic approach that utilises data from protein expression microarrays to explore the mechanism(s) by which SU^1349^ treatment affects human PC3 cell induced bone cell activity. Quantitative analysis of levels of PC3 cell-secreted factors in conditioned medium revealed that exposure to SU^1349^ (1.0 µM) is associated with dysregulation of a subset of 22 tumour-derived human cytokines and chemokines. As shown in Fig. 6A, SU^1349^ downregulated 21 (16 of which by 20% or more) and upregulated 1 factor that have previously been found to be involved in the regulation of prostate cancer, inflammation, angiogenesis and immunity (refer to supplementary Table S1). Further evaluation of the role of these secreted proteins in prostate cancer and bone metabolism revealed that all 22 factors identified are likely to be implicated in the disruptive effects of SU^1349^ on PC3 cell-induced osteoclast and osteoblast changes observed in our models (Fig. 6B and C).

## Discussion

Metastasis is a leading cause of death in advanced prostate cancer patients [53–55]. Bone metastases of prostatic origin are complex, multifaceted processes that result in the development of both osteosclerotic and osteolytic lesions [21, 23]. Genetic and pharmacological studies by a number of groups, including ours, have previously reported that inhibition of NF-kB at the level of IKK_α/β_ or IKK_e_ suppresses osteoclastogenesis and reduced bone loss caused by oestrogen deficiency, inflammation, and metastasis [30, 31, 56, 57]. Inhibition of the IKK/NF-kB axis also promotes new bone formation by enhancing osteoblast differentiation and maturation [30, 58–60]. Osteoclasts and osteoblasts play a vital role in the initiation and progression of secondary prostate cancer in bone [18–21]. Using the TRAMP model of mouse prostate cancer, Luo et al. showed that genetic inactivation of IKK_α_ inhibited prostate cancer metastasis [15]. Considering that TRAMP mice do not develop skeletal metastasis [61], the effect of IKK_α_ inhibition on prostate cancer-induced bone disease remained poorly understood. Thus, we utilised the recently discovered class of IKK_α_ inhibitors [42] to test the hypothesis that pharmacological inhibition of IKK_α_ is of therapeutic value in the treatment of prostate cancer-induced bone disease.

Osteolysis is the major cause of skeletal-related events associated with advanced prostate cancer [20, 22]. Using ex vivo and in vivo models of prostate cancer—bone cell interactions, we showed that treatment with the potent anti-tumour, and highly selective IKK inhibitor SU^1349^ enhanced bone volume in mouse calvaria ex vivo and trabecular bone in vivo. These effects were found to be accompanied by significant inhibition of osteoclast number and activity, thereby suggesting anti-resorptive properties for this agent. These findings are novel; but are in agreement with previous studies in models of benign bone disease that showed that IKK_α_ inhibition suppresses osteoclast number and size [37, 38, 62], and protects against trabecular bone loss following oestrogen deficiency [62]. Interestingly, mice treated with SU^1349^ exhibited significant increase in osteoblast number and activity and signs of formation of ectopic, disorganised bone—a feature of secondary prostate cancer in bone that contributes in part to bone fragility [63–65]. Microarray analysis of secreted proteins in conditioned medium from human PC3 cells treated with SU^1349^ detected significant changes in the levels of a cocktail of tumour-derived NF-κB-mediated osteolytic and osteoblastic factors (Fig. 6B and Table S1). Thus, it is likely that IKK_α_ inhibition by SU^1349^ orchestrated the expression of a set of PC3-derived factors that function cooperatively to alter the balance between osteoblasts and osteoclasts in our models.

To explore how SU^1349^ influences osteoblast function and bone formation in our models, we examined its effects on the osteoblast-rich cortical compartment of mice. This investigation revealed that administration of this agent in vivo caused cortical bone porosity and loss. While we these changes were significant, we were unable to perform biomechanical testing, in particular three-point bending, to confirm a functional increase in fracture risk. This remains an important area for future investigation. Nonetheless, histological analysis of the endocortical bone surface supported, at least in part, this finding and showed that SU^1349^ reduced the number and activity of osteoblasts, and enhanced osteoclastogenesis. In skeletal metastasis, different regions of bone exhibit varying numbers of tumour cells, metabolic and turnover rates, and vascularization. Therefore, it is expected to see an agent such as SU^1349^ exerting differential effects on osteoblasts and bone turnover at different compartments of bone through both enhanced and inhibited osteoblast differentiation, maturation, and to form bone nodules in vitro. Furthermore, the differential response between trabecular and cortical compartments is likely due to the distinct microenvironments and vascular supplies of these bone envelopes. Trabecular bone is highly metabolically active and responsive to changes in the hematopoietic/immune niche, whereas cortical bone is more reliant on osteoblast-lined periosteal and endosteal surfaces. It is plausible that IKK_α_ inhibition by SU1349 differentially affects NF-κB RelB signalling in cortical osteoblasts versus trabecular osteoblasts, or alters the RANKL/OPG ratio in a compartment-specific manner. In fact, differential regulation of osteoblasts and bone volume by perturbing non-canonical NF-κB signalling has been previously observed. Yao et. al. reported in 2014 that mice deficient in RelB exhibited increased osteoblast differentiation and trabecular, but not cortical, bone volume [66]. In contrast, Furuya and colleagues observed that inhibition of RANKL-related IKK_α/β_ activation in mice increased cortical, but not trabecular, bone formation and enhanced osteoblast differentiation [67]. In vitro, the biphasic effect observed on osteoblasts—stimulation at 0.01 µM and inhibition at 0.10 µM—is consistent with the concentration-dependent selectivity profile of SU1349. At lower concentrations (0.01 µM), SU1349 is highly selective for IKK_α_ over IKK_β_ (Ki selectivity ratio > 300-fold; [42]), potentially permitting some degree of non-canonical NF-κB signaling that supports osteoblast differentiation. At higher concentrations (0.10–1.0 µM), the compound may exhibit ‘off-target’ effects on IKK_β_ or other kinases, leading to suppression of both canonical and non-canonical signaling, thereby inhibiting osteoblast maturation.

Most IKK-NF-kB inhibitors that have been developed towards clinical use have been predominantly selective for IKK_β_ and so target canonical NF-kB signalling [39]. However, IKK_β_ manipulation has been found to exert no effect on prostate cancer metastasis [16]. Here, we report that cancer-specific inhibition of IKK_α_ by gene knockdown and administration of SU^1349^ inhibited the migratory and invasive ability of human PC3 cells in vitro, and SU^1349^ administration in mice showed a non-significant trend in reducing the metastatic spread of these cells (Fig. S5). The lack of significant reduction in overt metastasis in vivo, despite strong anti-migratory effects in vitro, may reflect the redundancy of metastatic drivers in the model utilised, the complex nature of the bone marrow microenvironment, and/or the short duration of treatment (12 days) in the in vivo protocol. Nevertheless, these present findings support our hypothesis and agree with previous studies that showed genetic inactivation [15] and pharmacological inhibition [68] of IKK_α_ inhibited soft tissue metastases in the TRAMP model.

IKK_α_ plays a role in the activation of canonical and non-canonical NFκB signalling [9, 69, 70], and itself translocates to the nucleus where it activates the expression of several NFkB-dependent genes [71, 72]. Our in vitro mechanistic experiments conducted in bone cells, namely osteoclasts and osteoblasts, suggest that SU^1349^ did indeed inhibit both canonical IkB and non-canonical P-100 activation; despite displaying higher selectivity towards IKK_α_ over IKK_β_ (Table 1). This contrasts with the recent report by Mackay and co-workers [42] that SU^1349^ selectively inhibited p100 phosphorylation via IKK_α_ in PC3M cells, with no impact on three pharmacodynamic markers of IKK_β_-mediated signalling at 10 µM (TNFα-stimulated p65 and p105 phosphorylation or reversal of IκBα degradation: see Table 1; Fig. 15 in [42]. However, this may be due to cross-talk between the two pathways under the conditions deployed to explore the dynamic interactions between osteoblast, osteoclast and PC3 cells, or it may suggest some degree of redundancy in regulating NF-kB signalling between the two isoforms. Thus, further studies are required to understand the relative contribution of the acute agonist-mediated activation of IKK_α_ versus IKK_β_ in driving the phenotypic changes in the bone and prostate cancer cell types studied here, with a comparative analysis of SU^1349^ with an IKK_β_-selective pharmacological inhibitor such as BIX02514. It is also possible that inhibition of both canonical and non-canonical NFkB activation in the immuno-compromised mouse model used might have contributed, at least in part, to the observed non-significant trend of increased cachexia in SU^1349^ treated mice bearing human PC3 cells for 35 days (Fig. S4). Future work should therefore investigate the effects of different dosing regimens for SU^1349^—other than 20 mg/kg/daily—immune-competent mouse models of prostate cancer [73].

## Conclusion

Our present study is the first to show that a selective IKK_α_ inhibitor, namely SU^1349^, reduced the ability of human PC3 cells to grow, migrate and invade in vitro, to induce osteolysis ex vivo, and to cause trabecular bone loss in mice. However, SU^1349^ caused cortical bone loss and failed to significantly reduce overt metastasis and cachexia in the immuno-compromised mice bearing the human PC3 cells. It is important to note that these studies were conducted in immunocompromised BALB/c nu/nu mice, which lack a functional T-cell compartment. Given that NF-κB signaling is central to both innate and adaptive immunity, future studies in immunocompetent models (such as syngeneic mouse models or humanized mice) are essential to determine the net effect of IKK_α_ inhibition on the immune-mediated control of prostate cancer growth in bone. These studies should also assess whether lower, less frequent dosing regimens might preserve the trabecular protective effect while mitigating cortical bone loss. That said, it is important to note that there are no clinically approved selective IKK_α_ inhibitors to be tested in cancer patients. Considering that a pilot study has already demonstrated that a member of this family of IKK_α_ inhibitors (subcutaneous injection, 50 mg/kg/8-days [74]) has been shown to reduce tumour growth in mice bearing human PC3 cells, our present findings—when combined with previous studies in models of prostate cancer [9, 13, 15–17] and bone metabolism [36–38]—suggest that SU^1349^ can be of therapeutic value in the treatment of osteolysis associated with advanced, difficult-to-treat secondary prostate cancer in bone. However, cortical bone loss may limit its long-term usefulness as an osteoprotective agent.

## Supplementary Information

Below is the link to the electronic supplementary material.

Supplementary Material 1

## Abbreviations

Alk Phos: Alkaline phosphatase
ALZ: Alizarin red
ANOVA: Analysis of variance
BM: Bone marrow
BT: Osteotropic
BV: Bone volume
C5/C5a: Complement component C5/C5a
CD40L: Cluster of differentiation 40 ligand
DMSO: Dimethyl sulfoxide
Emmprin: Extracellular matrix metalloproteinase inducer (aka. CD147)
ENA-78: Epithelial-neutrophil activating peptide (aka. CXCL5)
FCS: Fetal calf serum
GDF-15: Growth differentiation Factor 15
IKK: IκB kinase
IL1β: Interleukin 1 beta
IP: Intraperitoneal injection
LCN2: Lipocalin-2
LPS: Lipopolysaccharide
Luc: Luciferase
M-CSF: Macrophage colony-stimulating factor
MCP-1: Monocyte chemotactic protein-1 aka. CCL2
MEM: Minimum essential mediums.microCT, micro–computed tomography
MIP-3β: Macrophage inflammatory protein 3 beta (aka. CCL19)
mm: Millimeter
NFkB: Nuclear factor kappa-B
OPN: Osteopontin
PBS: Phosphate buffered saline
RANK: Receptor activator of NFkB
RANKL: RANK ligand
RBP-4: Retinol binding protein 4
SD: Standard deviation
TGF-α: Transforming growth factor-α
TNFα: Tumour necrosis factor alpha
TRAcP: Tartrate-resistant acid phosphatase
TSP-1: Thrombospondin-1
uPAR: Urokinase plasminogen activator receptor
