# Supplementary material for: Differential Effects of a First-in-Class IKKα Inhibitor on Trabecular and Cortical Bone in a Human Prostate Cancer Xenograft Model
Source: Calcif Tissue Int. 2026 Jul 25;117(1):122. doi: 10.1007/s00223-026-01558-y (PMC13401539; doi:10.1007/s00223-026-01558-y)
Supplement: Supplementary file 1 — Supplementary Material 1 [file 223_2026_1558_MOESM1_ESM.pdf]

Appendix A:

Supplementary materials

I. Figures 1
II. Tables 7
III. Supplementary references for Table S2 and Fig. 6 12

I. FIGURES

Figure S1.

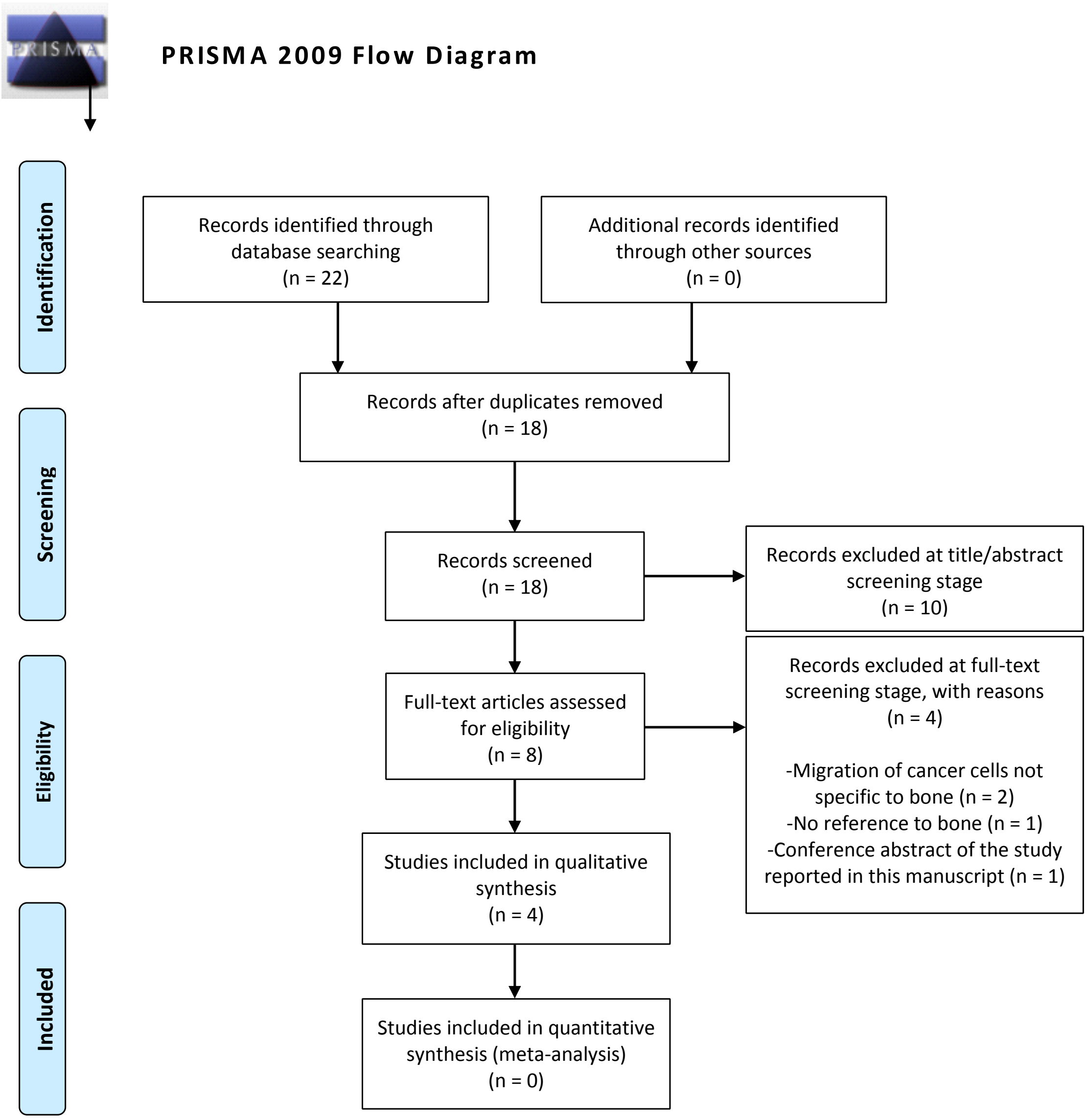

Figure S1. Systematic Reviews and Meta-analysis (PRISMA) flow diagram of evidence search and study selection process. n denotes number of articles.

Figure S2.

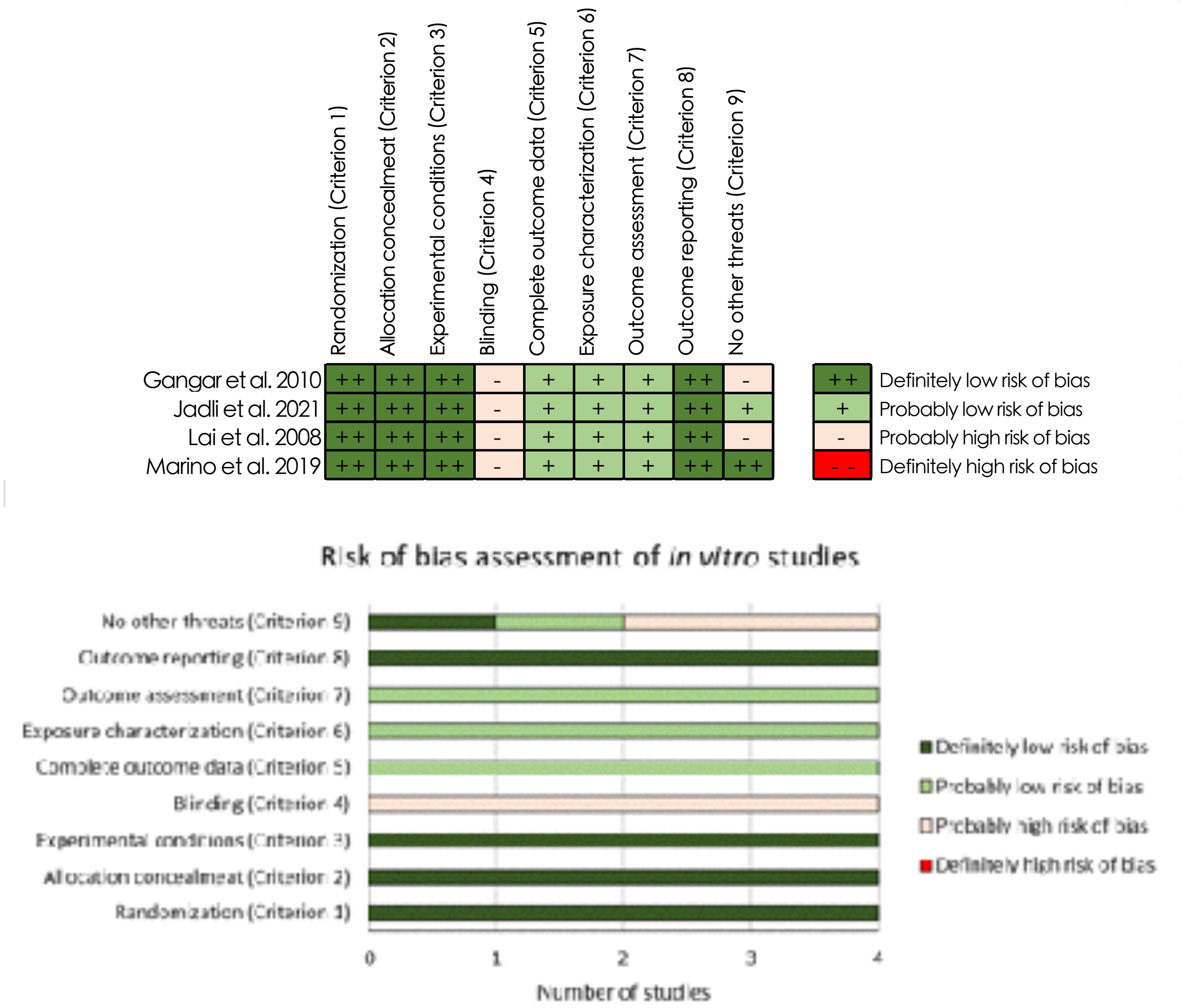

	Randomization (Criterion 1)	Allocation concealmeat (Criterion 2)	Experimental conditions (Criterion 3)	Blinding (Criterion 4)	Complete outcome data (Criterion 5)	Exposure characterization (Criterion 6)	Outcome assessment (Criterion 7)	Outcome reporting (Criterion 8)	No other threats (Criterion 9)
Gangar et al. 2010	++	++	++	-	+	+	+	++	-
Jadli et al. 2021	++	++	++	-	+	+	+	++	+
Lai et al. 2008	++	++	++	-	+	+	+	++	-
Marino et al. 2019	++	++	++	-	+	+	+	++	++

Figure S2. Risk of bias (RoB) assessment for systematic review included studies (OHAT RoB tool). Out of the 9 criteria only 'Blinding of research personnel during the study' (criterion 4) was scored as 'Probably high risk' across all in vitro studies. This was of no concern however, since it is rather uncommon for researchers to be blinded when performing in vitro experiments. For all remaining criteria, apart from criterion 9 (No other threats), all studies were considered 'Definitely low risk' or 'Probably low risk'. For criterion 9 (No other threats), 2 studies were considered 'Probably high risk', because of inappropriate statistical methods and/or because no statement regarding conflict of interest was provided. The overall risk of bias for all in vitro studies was 'Probably low' to 'Definitely low' (i.e., Tier 1 on a 3-tier system),and therefore, no study was excluded solely based on their quality.

Figure S3.

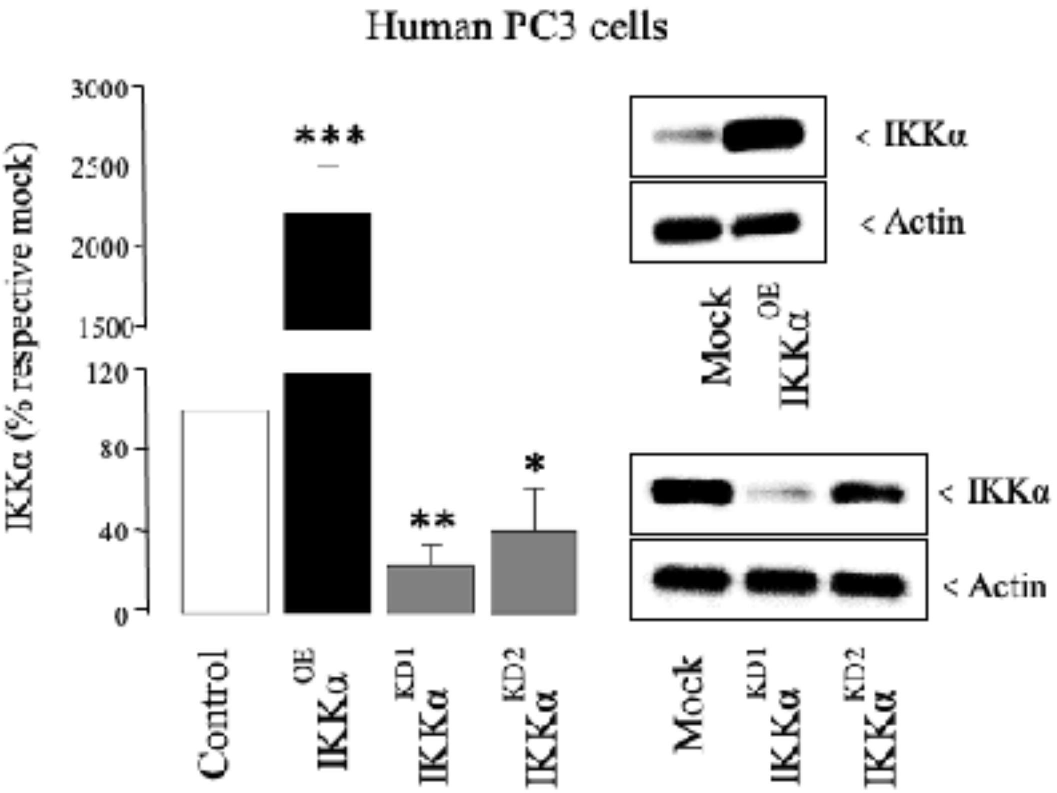

Figure S3 (*related to Figure 1*). Successful knockdown and over-expression of IKK$_\alpha$ in human PC3 cells. *In vitro* Western blot expression of IKKα in human PC3 cells overexpressing (IKK$_\alpha^{OE}$) and deficient in (IKK$_\alpha^{KD1}$ and ^{KD2}) IKK$_\alpha$ and their mock control cultured in serum-enriched complete media (FCS, 20% v/v). Values in graphs are mean (expressed as % of vehicle control) ± standard deviation (s.d.) and are obtained from 3 independent experiments. * $p < 0.05$; ** $p < 0.01$; *** $p < 0.001$ from vehicle or mock control.

Figure S4.

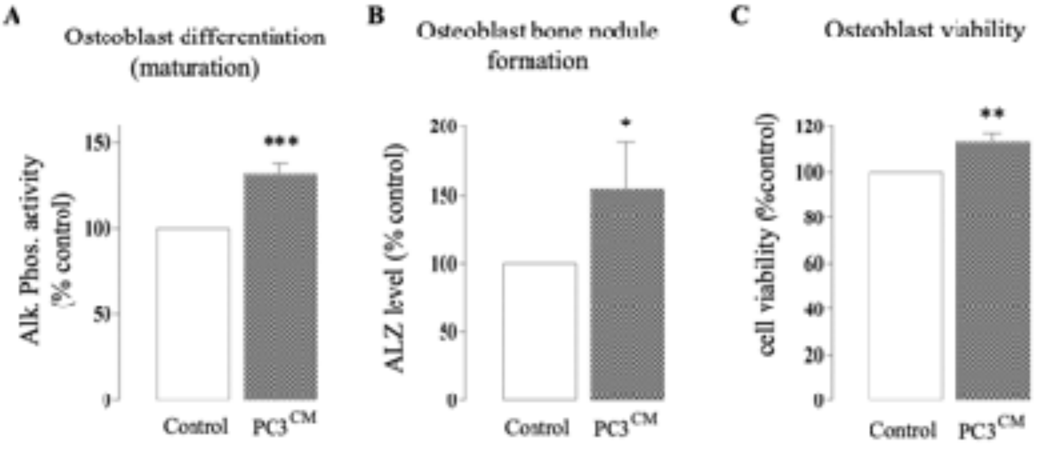

Figure S4 (*related to Figure 2*). PC3-derived factors enhance *in vitro* growth, differentiation and bone nodule formation in human osteoblasts. A. *In vitro* differentiation of human Saos-2 pre-osteoblasts in the absence and presence of conditioned medium (CM, 20% v/v) from human PC3 cells for 21 days, as assessed by alkaline phosphatase (Alk. Phos.) assay. **B.** *In vitro* bone nodule formation in cultures of human Saos-2 pre-osteoblasts from the experiment described in panel **A**, as assessed by Alazarin Red (ALZ) assay. **C.** *In vitro* cell viability in cultures of human Saos-2 pre-osteoblasts from the experiment described in panels **A-B**, as assessed by AlamarBlue assay. Values in graphs are mean (expressed as % of vehicle control) ± standard deviation (s.d.) and are obtained from 3 independent experiments. ** $p < 0.01$; *** $p < 0.001$ from vehicle or mock control.

Figure S5.

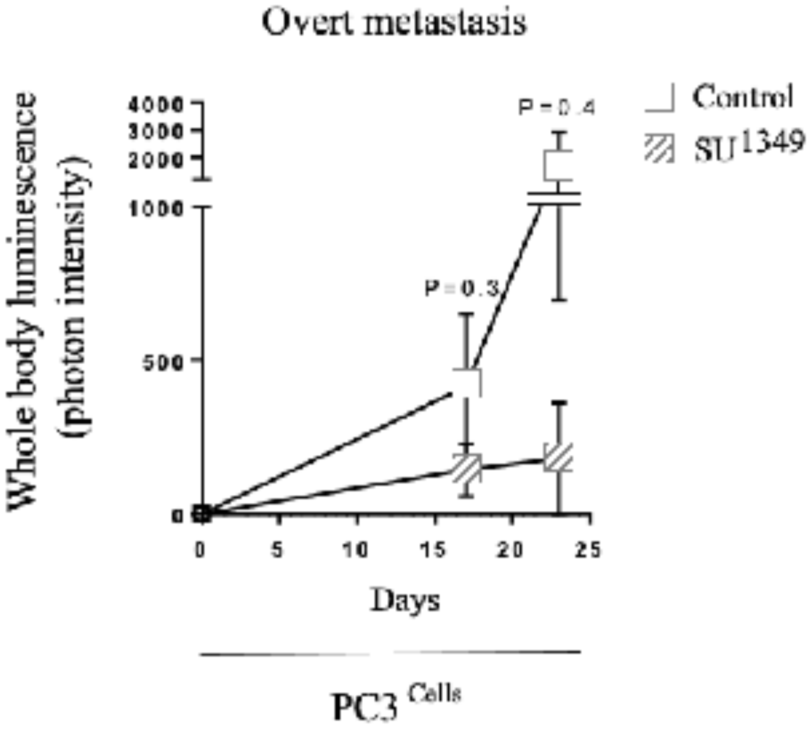

Figure S5 (*related to Figures 4 and 5*). Administration of SU1349 shows a non-significant trend towards reduced overt metastasis in mice bearing human PC3 cells. Quantification of whole-body luminescence in 8 weeks old adult BALB/c-nu/nu athymic mice following intra-cardiac injection of human PC3 cells and treatment with the selective IKK$_{\alpha}$ inhibitor SU^{1349} (n=16, 20mg/kg/3times-weekly) or vehicle (n=13) for 12 days. Values are mean ± SD; p = 0.3 and 0.4 compared to vehicle treated group (control).

Figure S6.

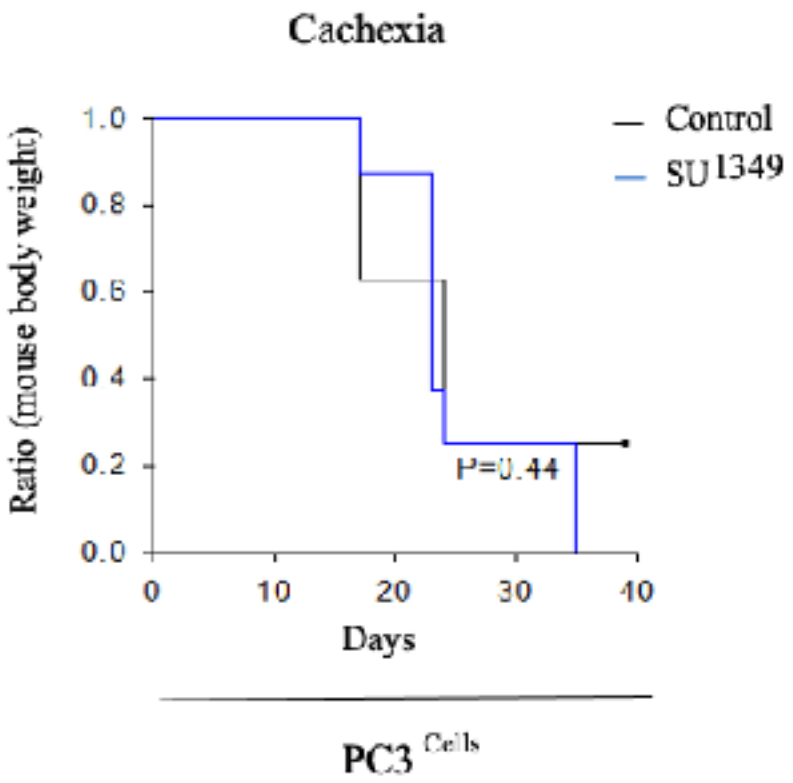

Figure S6 (*related to Figures 4 and 5***). Administration of SU1349 has no significant effect on cachexia in mice bearing human PC3 cells**. Immuno-deficient BALB/c mice (8 mice per group) were intra-cardiacally injected with luciferase expressing human PC3 cells and treated with SU1349 (20mg/kg/3times-weekly) or vehicle control. The graph shows the effects of SU1349 or vehicle control treatment on cachexia in BALB/c mice as assessed by daily monitoring of body weight; cachexia is expressed as a ratio of mice weight measured during the experiment normalised against weight of mice at the start of the experiments. Values are mean ± SD; $p = 0.44$ compared to vehicle treated group (control).

II. SUPPLEMENTARY TABLES

Table S1 List of search strategies of Web of Science, MEDLINE and EMBASE.

Medline and EMBASE	Ovid MEDLINE®
1. exp Prostatic Neoplasms/	1. TS=(prostate Cancer* OR prostatic cancer* OR prostate neoplasm* OR prostatic neoplasm* OR cancer of prostate OR cancer of the prostate)
2. prostate cancer*.mp.	2. TS=(bone metastasis OR metastasis to bone OR secondary bone tumo$rs OR metastatic bone OR metastatic bone cancer OR osseous metastasis OR osseous metastatic disease OR skeletal metastasis)
3. prostatic cancer*.mp.	3. TS=(osteoblasts OR osteoclasts OR bone formation OR ossification OR bone resorption OR bone loss OR bone remodelling OR trabecular bone* OR cancellous bone* OR spongy bone* OR cortical bone* OR compact bone* OR [illegible]
4. prostate neoplasm*.mp.	4. #2 OR #3
5. prostatic neoplasm*.mp.	5. TS=(inhibitory-kB kinase a OR inhibitory kB kinase a OR inhibitory-kappa B kinase alpha OR Inhibitory kappa B kinase alpha OR IkappaB Kinase alpha OR IKK alpha, IKK_{α} OR IKK_{α} OR IKK-alpha)
6. cancer of prostate.mp.	6. #5 AND #4 AND #1
7. cancer of the prostate.mp.	
8. 1 or 2 or 3 or 4 or 5 or 6 or 7	
9. exp Bone Neoplasms/	
10. bone metastasis.mp.	
11. metastasis to bone.mp.	
12. secondary bone tumo$rs.mp.	
13. metastatic bone.mp.	
14. metastatic bone cancer.mp.	
15. osseous metastasis.mp.	
16. osseous metastatic disease.mp.	
17. skeletal metastasis.mp.	

18. 9 or 10 or 11 or 12 or 13 or 14 or 15 or 16 or 17	
19. exp Osteoblasts/	
20. exp Osteoclasts/	
21. (bone formation or ossification).mp.	
22. (bone resorption or bone loss).mp.	
23. bone remodelling.mp.	
24. (trabecular bone* or cancellous bone* or spongy bone*).mp.	
25. (cortical bone* or compact bone* or substantia compacta).mp.	
26. 19 or 20 or 21 or 22 or 23 or 24 or 25	
27. 18 or 26	
28. inhibitory-kB kinase a.mp.	
29. inhibitory kB kinase a.mp.	
30. inhibitory-kappa B kinase alpha.mp.	
31. Inhibitory kappa B kinase alpha.mp.	
32. IkappaB Kinase alpha.mp.	
33. (IKK alpha, IKK_{α} or IKKalpha or IKK-alpha).mp.	
34. 28 or 29 or 30 or 31 or 32 or 33	
35. 8 and 27 and 34	

Table S2 (related to Figure 6, panel A and B). Summary table for effects of SU1349 on differentially regulated factors on prostate cancer cells, osteoclasts and osteoblasts and adipocytes. Levels of human cytokines and chemokines in conditioned medium from human PC3 prostate cancer cell line as measured by Proteome Profiler Human XL Cytokine Array Kit. Abbreviation; Ref, references. Effects on cells (reported in the literature) are indicated by: ↑ = positive; ↓ = negative; ↑↓ = positive and/or negative; ↔ = an ambiguous association; -- = no association found. References for effects are also included.

Protein	Prostate cancer	Osteoclast	Osteoblast	Adipocytes	Refs
**MCP-1**	↑	↑	↔	↓	[1-4]
**IL-1β**	↑	↑	↓	↓	[5-9]
**ENA-78**	↑	↑	↔	↓	[10-15]
**IL-2**	↑	↑	_	↑	[16-19]
**IL-1α**	↑	↑	↓	↓	[20-24]
**IL-24**	↓	_	↑↓	↔	[24-28]
**OPN**	↔	↑	↓	↓	[29-33]
**RBP-4**	↑	↔	↑↓	↓	[34] [35-38]
**IL-8**	↑	↑	↔	↓	[39-42]
**IL-17A**	↑	↓	↑↓	↓	[43-48]
**MIP-3β**	↑*	↑	↑	↔	[49-52] ***retracted**
**IL-11**	↑	↑	↑↓	↓	[53-56]
**LCN2**	↑	↓	↓	↑	[57-60]
**TGF-α**	↑	↑	↑	↑↓	[61-64]
**uPAR**	↑	↑	↓	↑	[65-67]
**Adipsin**	↔	_	_	↔	[68]
**C5/C5a**	↑	↑	↔	↓	[69-71]
**IL-33**	↓	↓	↑	↑	[72-74]
**GDF-15**	↑↓	↑	↓	↓	[75-78]
**TSP-1**	↑	↑	↓	↑↓	[79-82]
**Angiogenin**	↑	↓	_	_	[83, 84]
**Emmprin**	↑	↑	↑	_	[85-88]

Abbreviations: **MCP-1** (Monocyte chemotactic protein-1 aka. CCL2); **ENA-78** (Epithelial-neutrophil activating peptide; aka. CXCL5); **OPN** (Osteopontin); **RBP-4** (Retinol binding protein 4); **MIP-3β** (Macrophage inflammatory protein 3 beta aka. CCL19); **LCN2**

(Lipocalin-2); **TGF-α** (Transforming growth factor- α); **uPAR** (Urokinase plasminogen activator receptor); **C5/C5a** (Complement component C5/C5a); **GDF-15** (Growth Differentiation Factor 15); **TSP-1** (Thrombospondin-1); **Emmprin** (Extracellular matrix metalloproteinase inducer aka. CD147).

REFERENCES (related to Table 21 and Fig. 6)

[1] Y. Lu, Q. Chen, E. Corey, W. Xie, J. Fan, A. Mizokami, J. Zhang, Activation of MCP-1/ CCR2 axis promotes prostate cancer growth in bone, Clinical & experimental metastasis, 26 (2009) 161-169.
[2] X. Li, L. Qin, M. Bergenstock, L.M. Bevelock, D.V. Novack, N.C. Partridge, Parathyroid hormone stimulates osteoblastic expression of MCP-1 to recruit and increase the fusion of pre/osteoclasts, The Journal of biological chemistry, 282 (2007) 33098-33106.
[3] Y. Lu, G. Xiao, D.L. Galson, Y. Nishio, A. Mizokami, E.T. Keller, Z. Yao, J. Zhang, PTHrP-induced MCP-1 production by human bone marrow endothelial cells and osteoblasts promotes osteoclast differentiation and prostate cancer cell proliferation and invasion in vitro, International journal of cancer. Journal international du cancer, 121 (2007) 724-733.
[4] H. Kanda, S. Tateya, Y. Tamori, K. Kotani, K. Hiasa, R. Kitazawa, S. Kitazawa, H. Miyachi, S. Maeda, K. Egashira, M. Kasuga, MCP-1 contributes to macrophage infiltration into adipose tissue, insulin resistance, and hepatic steatosis in obesity, The Journal of clinical investigation, 116 (2006) 1494-1505.
[5] E. Voronov, D.S. Shouval, Y. Krelin, E. Cagnano, D. Benharroch, Y. Iwakura, C.A. Dinarello, R.N. Apte, IL-1 is required for tumor invasiveness and angiogenesis, Proceedings of the National Academy of Sciences of the United States of America, 100 (2003) 2645-2650.
[6] B. Lee, T.H. Kim, J.B. Jun, D.H. Yoo, J.H. Woo, S.J. Choi, Y.H. Lee, G.G. Song, J. Sohn, K.H. Park-Min, L.B. Ivashkiv, J.D. Ji, Direct inhibition of human RANK+ osteoclast precursors identifies a homeostatic function of IL-1beta, Journal of immunology, 185 (2010) 5926-5934.
[7] R.S. Taichman, P.V. Hauschka, Effects of interleukin-1 beta and tumor necrosis factor-alpha on osteoblastic expression of osteocalcin and mineralized extracellular matrix in vitro, Inflammation, 16 (1992) 587-601.
[8] C. Bing, Is interleukin-1beta a culprit in macrophage-adipocyte crosstalk in obesity?, Adipocyte, 4 (2015) 149-152.
[9] D.P. Trebec-Reynolds, I. Voronov, J.N. Heersche, M.F. Manolson, IL-1alpha and IL-1beta have different effects on formation and activity of large osteoclasts, Journal of cellular biochemistry, 109 (2010) 975-982.
[10] L.A. Begley, S. Kasina, R. Mehra, S. Adsule, A.J. Admon, R.J. Lonigro, A.M. Chinnaiyan, J.A. Macoska, CXCL5 promotes prostate cancer progression, Neoplasia, 10 (2008) 244-254.
[11] K. Sundaram, D.S. Rao, W.L. Ries, S.V. Reddy, CXCL5 stimulation of RANK ligand expression in Paget's disease of bone, Lab Invest, 93 (2013) 472-479.
[12] H. Roca, J.D. Jones, M.C. Purica, S. Weidner, A.J. Koh, R. Kuo, J.E. Wilkinson, Y. Wang, S. Daignault-Newton, K.J. Pienta, T.M. Morgan, E.T. Keller, J.E. Nor, L.D. Shea, L.K. McCauley, Apoptosis-induced CXCL5 accelerates inflammation and growth of prostate tumor metastases in bone, The Journal of clinical investigation, 128 (2018) 248-266.
[13] Y.L. Hsu, M.F. Hou, P.L. Kuo, Y.F. Huang, E.M. Tsai, Breast tumor-associated osteoblast-derived CXCL5 increases cancer progression by ERK/MSK1/Elk-1/snail signaling pathway, Oncogene, 32 (2013) 4436-4447.
[14] C. Chavey, L. Fajas, CXCL5 drives obesity to diabetes, and further, Aging, 1 (2009) 674-677.
[15] S.M. Kabir, E.S. Lee, D.S. Son, Chemokine network during adipogenesis in 3T3-L1 cells: Differential response between growth and proinflammatory factor in preadipocytes vs. adipocytes, Adipocyte, 3 (2014) 97-106.
[16] A. Belldegrun, C.L. Tso, A. Zisman, J. Naitoh, J. Said, A.J. Pantuck, A. Hinkel, J. deKernion, R. Figlin, Interleukin 2 gene therapy for prostate cancer: phase I clinical trial and basic biology, Hum Gene Ther, 12 (2001) 883-892.
[17] W.L. Ries, M.C. Seeds, L.L. Key, Interleukin-2 stimulates osteoclastic activity: increased acid production and radioactive calcium release, J Periodontal Res, 24 (1989) 242-246.
[18] S.A. Vielma, R.L. Klein, C.A. Levingston, M.R. Young, Adipocytes as immune regulatory cells, International immunopharmacology, 16 (2013) 224-231.

[19] J.Y. Huh, Y.J. Park, M. Ham, J.B. Kim, Crosstalk between Adipocytes and Immune Cells in Adipose Tissue Inflammation and Metabolic Dysregulation in Obesity, Mol Cells, 37 (2014) 365-371.
[20] M. Ricote, I. Garcia-Tunon, F.R. Bethencourt, B. Fraile, R. Paniagua, M. Royuela, Interleukin-1 (IL-1alpha and IL-1beta) and its receptors (IL-1RI, IL-1RII, and IL-1Ra) in prostate carcinoma, Cancer, 100 (2004) 1388-1396.
[21] N. Tani-Ishii, A. Tsunoda, T. Teranaka, T. Umemoto, Autocrine regulation of osteoclast formation and bone resorption by IL-1 alpha and TNF alpha, Journal of dental research, 78 (1999) 1617-1623.
[22] N. Tanabe, E. Ito-Kato, N. Suzuki, A. Nakayama, B. Ogiso, M. Maeno, K. Ito, IL-1alpha affects mineralized nodule formation by rat osteoblasts, Life Sci, 75 (2004) 2317-2327.
[23] J.Y. Um, H.K. Rim, S.J. Kim, H.L. Kim, S.H. Hong, Functional polymorphism of IL-1 alpha and its potential role in obesity in humans and mice, PloS one, 6 (2011) e29524.
[24] M. Sauane, Z.Z. Su, P. Gupta, I.V. Lebedeva, P. Dent, D. Sarkar, P.B. Fisher, Autocrine regulation of mda-7/IL-24 mediates cancer-specific apoptosis, Proceedings of the National Academy of Sciences of the United States of America, 105 (2008) 9763-9768.
[25] K.M. Lee, H.A. Kang, M. Park, H.Y. Lee, H.R. Choi, C.H. Yun, J.W. Oh, H.S. Kang, Interleukin-24 attenuates beta-glycerophosphate-induced calcification of vascular smooth muscle cells by inhibiting apoptosis, the expression of calcification and osteoblastic markers, and the Wnt/beta-catenin pathway, Biochemical and biophysical research communications, 428 (2012) 50-55.
[26] T.W. Kragstrup, M.N. Andersen, B. Schiottz-Christensen, A.G. Jurik, M. Hvid, B. Deleuran, Increased interleukin (IL)-20 and IL-24 target osteoblasts and synovial monocytes in spondyloarthritis, Clin Exp Immunol, 189 (2017) 342-351.
[27] T.W. Kragstrup, S.R. Greisen, M.A. Nielsen, C. Rhodes, K. Stengaard-Pedersen, M.L. Hetland, K. Horslev-Petersen, P. Junker, M. Ostergaard, M. Hvid, T. Vorup-Jensen, W.H. Robinson, J. Sokolove, B. Deleuran, The interleukin-20 receptor axis in early rheumatoid arthritis: novel links between disease-associated autoantibodies and radiographic progression, Arthritis Res Ther, 18 (2016) 61.
[28] A.L. Strong, D.T. Pei, C.G. Hurst, J.M. Gimble, M.E. Burow, B.A. Bunnell, Obesity Enhances the Conversion of Adipose-Derived Stromal/Stem Cells into Carcinoma-Associated Fibroblast Leading to Cancer Cell Proliferation and Progression to an Invasive Phenotype, Stem Cells Int, 2017 (2017) 9216502.
[29] J.W. Thoms, A. Dal Pra, P.H. Anborgh, E. Christensen, N. Fleshner, C. Menard, K. Chadwick, M. Milosevic, C. Catton, M. Pintilie, A.F. Chambers, R.G. Bristow, Plasma osteopontin as a biomarker of prostate cancer aggression: relationship to risk category and treatment response, British journal of cancer, 107 (2012) 840-846.
[30] T. Yamate, H. Mocharla, Y. Taguchi, J.U. Igietseme, S.C. Manolagas, E. Abe, Osteopontin expression by osteoclast and osteoblast progenitors in the murine bone marrow: demonstration of its requirement for osteoclastogenesis and its increase after ovariectomy, Endocrinology, 138 (1997) 3047-3055.
[31] W. Huang, B. Carlsen, G. Rudkin, M. Berry, K. Ishida, D.T. Yamaguchi, T.A. Miller, Osteopontin is a negative regulator of proliferation and differentiation in MC3T3-E1 pre-osteoblastic cells, Bone, 34 (2004) 799-808.
[32] M. Tardelli, K. Zeyda, V. Moreno-Viedma, B. Wanko, N.G. Grun, G. Staffler, M. Zeyda, T.M. Stulnig, Osteopontin is a key player for local adipose tissue macrophage proliferation in obesity, Mol Metab, 5 (2016) 1131-1137.
[33] M. Zeyda, K. Gollinger, J. Todoric, F.W. Kiefer, M. Keck, O. Aszmann, G. Prager, G.J. Zlabinger, P. Petzelbauer, T.M. Stulnig, Osteopontin Is an Activator of Human Adipose Tissue Macrophages and Directly Affects Adipocyte Function, Endocrinology, 152 (2011) 2219-2227.
[34] H. Uehara, T. Takahashi, K. Izumi, Induction of retinol-binding protein 4 and placenta-specific 8 expression in human prostate cancer cells remaining in bone following osteolytic tumor growth inhibition by osteoprotegerin, Int J Oncol, 43 (2013) 365-374.
[35] A. Ghanem, Y.H. Lu, T.Y. Cai, X.Z. Mu, Overexpression of RBP4 promotes proliferation, differentiation and mineralization of MC3T3-E1 (vol 10, pg 298, 2017), Int J Clin Exp Patho, 10 (2017) 8092-8092.

[36] M. Hogstrom, A. Nordstrom, P. Nordstrom, Retinol, retinol-binding protein 4, abdominal fat mass, peak bone mineral density, and markers of bone metabolism in men: the Northern Osteoporosis and Obesity (NO2) Study, Eur J Endocrinol, 158 (2008) 765-770.
[37] J. Norseen, T. Hosooka, A. Hammarstedt, M.M. Yore, S. Kant, P. Aryal, H. Maruyama, B.J. Kraus, A. Usheva, R.J. Davis, U. Smith, B.B. Kahn, RBP4 Inhibits Insulin Signaling in Adipocytes by Inducing Pro-Inflammatory Cytokines in Macrophages through a JNK- and TLR4-Dependent and Retinol-Independent Mechanism, Diabetes, 61 (2012) A85-A85.
[38] V.D. Leitch, P.P. Dwivedi, P.J. Anderson, B.C. Powell, Retinol-binding protein 4 downregulation during osteogenesis and its localization to non-endocytic vesicles in human cranial suture mesenchymal cells suggest a novel tissue function, Histochem Cell Biol, 139 (2013) 75-87.
[39] B. Neveu, X. Moreel, M.P. Deschenes-Rompre, A. Bergeron, H. LaRue, C. Ayari, Y. Fradet, V. Fradet, IL-8 secretion in primary cultures of prostate cells is associated with prostate cancer aggressiveness, Res Rep Urol, 6 (2014) 27-34.
[40] M.S. Bendre, D.C. Montague, T. Peery, N.S. Akel, D. Gaddy, L.J. Suva, Interleukin-8 stimulation of osteoclastogenesis and bone resorption is a mechanism for the increased osteolysis of metastatic bone disease, Bone, 33 (2003) 28-37.
[41] K.M. Bussard, D.J. Venzon, A.M. Mastro, Osteoblasts Are a Major Source of inflammatory Cytokines in the Tumor Microenvironment of Bone Metastatic Breast Cancer, Journal of cellular biochemistry, 111 (2010) 1138-1148.
[42] C. Kobashi, S. Asamizu, M. Ishiki, M. Iwata, I. Usui, K. Yamazaki, K. Tobe, M. Kobayashi, M. Urakaze, Inhibitory effect of IL-8 on insulin action in human adipocytes via MAP kinase pathway, J Inflamm-Lond, 6 (2009).
[43] Q. Zhang, S. Liu, K.R. Parajuli, W. Zhang, K. Zhang, Z. Mo, J. Liu, Z. Chen, S. Yang, A. Wang, L. Myers, Z. You, Interleukin-17 promotes prostate cancer via MMP7-induced epithelial-to-mesenchymal transition, Oncogene, 36 (2017) 687-699.
[44] S. Kitami, H. Tanaka, T. Kawato, N. Tanabe, T. Katono-Tani, F. Zhang, N. Suzuki, Y. Yonehara, M. Maeno, IL-17A suppresses the expression of bone resorption-related proteinases and osteoclast differentiation via IL-17RA or IL-17RC receptors in RAW264.7 cells, Biochimie, 92 (2010) 398-404.
[45] M. Croes, F.C. Oner, D. van Neerven, E. Sabir, M.C. Kruyt, T.J. Blokhuis, W.J.A. Dhert, J. Alblas, Proinflammatory T cells and IL-17 stimulate osteoblast differentiation, Bone, 84 (2016) 262-270.
[46] Y. Qu, Q.Y. Zhang, S.Q. Ma, S. Liu, Z.Q. Chen, Z.F. Mo, Z.B. You, Interleukin-17A Differentially Induces Inflammatory and Metabolic Gene Expression in the Adipose Tissues of Lean and Obese Mice, Int J Mol Sci, 17 (2016).
[47] Z. Wang, Y. Jia, F. Du, M. Chen, X. Dong, Y. Chen, W. Huang, IL-17A Inhibits Osteogenic Differentiation of Bone Mesenchymal Stem Cells via Wnt Signaling Pathway, Med Sci Monit, 23 (2017) 4095-4101.
[48] M. Ahmed, S.L. Gaffen, IL-17 inhibits adipogenesis in part via C/EBPalpha, PPARgamma and Kruppel-like factors, Cytokine, 61 (2013) 898-905.
[49] C. Peng, K.L. Zhou, S.S. An, J. Yang, The effect of CCL19/CCR7 on the proliferation and migration of cell in prostate cancer, Tumor Biol, 36 (2015) 329-335.
[50] J. Lee, C. Park, H.J. Kim, Y.D. Lee, Z.H. Lee, Y.W. Song, H.H. Kim, Stimulation of osteoclast migration and bone resorption by C-C chemokine ligands 19 and 21, Experimental and Molecular Medicine, 49 (2017).
[51] Y. Qin, L.D. He, Z.J. Sheng, M.M. Yong, Y.S. Sheng, X.W. Dong, T.W. Wen, Z.Y. Ming, Increased CCL19 and CCL21 levels promote fibroblast ossification in ankylosing spondylitis hip ligament tissue, Bmc Musculoskel Dis, 15 (2014).
[52] F. Tourniaire, B. Romier-Crouzet, J.H. Lee, J. Marcotorchino, E. Gouranton, J. Salles, C. Malezet, J. Astier, P. Darmon, E. Blouin, S. Walrand, J.P. Ye, J.F. Landrier, Chemokine Expression in Inflamed Adipose Tissue Is Mainly Mediated by NF-kappa B, PloS one, 8 (2013).
[53] Y. Furuya, R. Nishio, A. Junicho, O. Nagakawa, H. Fuse, Serum interleukin-11 in patients with benign prostatic hyperplasia and prostate cancer, Int Urol Nephrol, 37 (2005) 69-71.

[54] O. Kudo, A. Sabokbar, A. Pocock, I. Itonaga, Y. Fujikawa, N.A. Athanasou, Interleukin-6 and interleukin-11 support human osteoclast formation by a RANKL-independent mechanism, Bone, 32 (2003) 1-7.
[55] T. Matsumoto, R. Kuriwaka-Kido, T. Kondo, I. Endo, S. Kido, Regulation of osteoblast differentiation by interleukin-11 via AP-1 and Smad signaling, Endocr J, 59 (2012) 91-101.
[56] D.C. Keller, X.X. Du, E.F. Srour, R. Hoffman, D.A. Williams, Interleukin-11 Inhibits Adipogenesis and Stimulates Myelopoiesis in Human Long-Term Marrow Cultures, Blood, 82 (1993) 1428-1435.
[57] M.C. Tung, S.C. Hsieh, S.F. Yang, C.W. Cheng, R.T. Tsai, S.C. Wang, M.H. Huang, Y.H. Hsieh, Knockdown of lipocalin-2 suppresses the growth and invasion of prostate cancer cells, The Prostate, 73 (2013) 1281-1290.
[58] H.J. Kim, H.J. Yoon, K.A. Yoon, M.R. Gwon, S. Jin Seong, K. Suk, S.Y. Kim, Y.R. Yoon, Lipocalin-2 inhibits osteoclast formation by suppressing the proliferation and differentiation of osteoclast lineage cells, Exp Cell Res, 334 (2015) 301-309.
[59] N. Rucci, M. Capulli, S.G. Piperni, A. Cappariello, P. Lau, P. Frings-Meuthen, M. Heer, A. Teti, Lipocalin 2: a new mechanoresponding gene regulating bone homeostasis, Journal of bone and mineral research : the official journal of the American Society for Bone and Mineral Research, 30 (2015) 357-368.
[60] J. Zhang, Y. Wu, Y. Zhang, D. Leroith, D.A. Bernlohr, X. Chen, The role of lipocalin 2 in the regulation of inflammation in adipocytes and macrophages, Molecular endocrinology, 22 (2008) 1416-1426.
[61] X.H. Liu, H.S. Wiley, A.W. Meikle, Androgens regulate proliferation of human prostate cancer cells in culture by increasing transforming growth factor-alpha (TGF-alpha) and epidermal growth factor (EGF)/TGF-alpha receptor, J Clin Endocrinol Metab, 77 (1993) 1472-1478.
[62] N. Takahashi, B.R. MacDonald, J. Hon, M.E. Winkler, R. Derynck, G.R. Mundy, G.D. Roodman, Recombinant human transforming growth factor-alpha stimulates the formation of osteoclast-like cells in long-term human marrow cultures, The Journal of clinical investigation, 78 (1986) 894-898.
[63] F.S. Panagakos, Transforming growth factor--alpha stimulates chemotaxis of osteoblasts and osteoblast-like cells in vitro, Biochem Mol Biol Int, 33 (1994) 643-650.
[64] S.C. Butterwith, C.D. Peddie, C. Goddard, Effects of transforming growth factor-alpha on chicken adipocyte precursor cells in vitro, The Journal of endocrinology, 134 (1992) 163-168.
[65] F. Margheri, S. D'Alessio, S. Serrati, M. Pucci, F. Annunziato, L. Cosmi, F. Liotta, R. Angeli, A. Angelucci, G.L. Gravina, N. Rucci, M. Bologna, A. Teti, B. Monia, G. Fibbi, M. Del Rosso, Effects of blocking urokinase receptor signaling by antisense oligonucleotides in a mouse model of experimental prostate cancer bone metastases, Gene Ther, 12 (2005) 702-714.
[66] F. Furlan, C. Galbiati, N.R. Jorgensen, J.E. Jensen, E. Mrak, A. Rubinacci, F. Talotta, P. Verde, F. Blasi, Urokinase plasminogen activator receptor affects bone homeostasis by regulating osteoblast and osteoclast function, Journal of bone and mineral research : the official journal of the American Society for Bone and Mineral Research, 22 (2007) 1387-1396.
[67] Y. Kanno, H. Matsuno, E. Kawashita, K. Okada, H. Suga, S. Ueshima, O. Matsuo, Urokinase-type plasminogen activator receptor is associated with the development of adipose tissue, Thromb Haemostasis, 104 (2010) 1124-1132.
[68] R. Ribeiro, C. Monteiro, R. Silvestre, A. Castela, H. Coutinho, A. Fraga, P. Principe, C. Lobato, C. Costa, A. Cordeiro-da-Silva, J.M. Lopes, C. Lopes, R. Medeiros, Human periprostatic white adipose tissue is rich in stromal progenitor cells and a potential source of prostate tumor stroma, Exp Biol Med, 237 (2012) 1155-1162.
[69] H. Nitta, Y. Wada, Y. Kawano, Y. Murakami, A. Irie, K. Taniguchi, K. Kikuchi, G. Yamada, K. Suzuki, J. Honda, M. Wilson-Morifuji, N. Araki, M. Eto, H. Baba, T. Imamura, Enhancement of Human Cancer Cell Motility and Invasiveness by Anaphylatoxin C5a via Aberrantly Expressed C5a Receptor (CD88), Clinical Cancer Research, 19 (2013) 2004-2013.
[70] A. Ignatius, P. Schoengraf, L. Kreja, A. Liedert, S. Recknagel, S. Kandert, R.E. Brenner, M. Schneider, J.D. Lambris, M. Huber-Lang, Complement C3a and C5a modulate osteoclast

formation and inflammatory response of osteoblasts in synergism with IL-1beta, Journal of cellular biochemistry, 112 (2011) 2594-2605.
[71] J. Phieler, K.J. Chung, A. Chatzigeorgiou, A. Klotzsche-von Ameln, R. Garcia-Martin, D. Sprott, M. Moisidou, T. Tzanavari, B. Ludwig, E. Baraban, M. Ehrhart-Bornstein, S.R. Bornstein, H. Mziaut, M. Solimena, K.P. Karalis, M. Economopoulou, J.D. Lambris, T. Chavakis, The Complement Anaphylatoxin C5a Receptor Contributes to Obese Adipose Tissue Inflammation and Insulin Resistance, Journal of immunology, 191 (2013) 4367-4374.
[72] I. Saranchova, J. Han, H. Huang, F. Fenninger, K.B. Choi, L. Munro, C. Pfeifer, I. Welch, A.W. Wyatt, L. Fazli, M.E. Gleave, W.A. Jefferies, Discovery of a Metastatic Immune Escape Mechanism Initiated by the Loss of Expression of the Tumour Biomarker Interleukin-33, Sci Rep-Uk, 6 (2016).
[73] H. Saleh, D. Eeles, J.M. Hodge, G.C. Nicholson, R. Gu, S. Pompolo, M.T. Gillespie, J.M.W. Quinn, Interleukin-33, a Target of Parathyroid Hormone and Oncostatin M, Increases Osteoblastic Matrix Mineral Deposition and Inhibits Osteoclast Formation in Vitro, Endocrinology, 152 (2011) 1911-1922.
[74] J.M. Han, D. Wu, H.C. Denroche, Y. Yao, C.B. Verchere, M.K. Levings, IL-33 Reverses an Obesity-Induced Deficit in Visceral Adipose Tissue ST2(+) T Regulatory Cells and Ameliorates Adipose Tissue Inflammation and Insulin Resistance, Journal of immunology, 194 (2015) 4777-4783.
[75] Y. Husaini, G.P. Lockwood, T.V. Nguyen, V.W.W. Tsai, M.G. Mohammad, P.J. Russell, D.A. Brown, S.N. Breit, Macrophage Inhibitory Cytokine-1 (MIC-1/GDF15) Gene Deletion Promotes Cancer Growth in TRAMP Prostate Cancer Prone Mice, PloS one, 10 (2015).
[76] M. Westhrin, S.H. Moen, T. Holien, A.K. Mylin, L. Heickendorff, O.E. Olsen, A. Sundan, I. Turesson, P. Gimsing, A. Waage, T. Standal, Growth differentiation factor 15 (GDF15) promotes osteoclast differentiation and inhibits osteoblast differentiation and high serum GDF15 levels are associated with multiple myeloma bone disease, Haematologica, 100 (2015) E511-E514.
[77] M. Yanagitai, T. Kitagawa, K. Okawa, H. Koyama, T. Satoh, Phenylenediamine derivatives induce GDF-15/MIC-1 and inhibit adipocyte differentiation of mouse 3T3-L1 Cells, Biochemical and biophysical research communications, 417 (2012) 294-298.
[78] Q. Ding, T. Mracek, P. Gonzalez-Muniesa, K. Kos, J. Wilding, P. Trayhurn, C. Bing, Identification of Macrophage Inhibitory Cytokine-1 in Adipose Tissue and Its Secretion as an Adipokine by Human Adipocytes, Endocrinology, 150 (2009) 1688-1696.
[79] V. Firlej, J.R.R. Mathieu, C. Gilbert, L. Lemonnier, J. Nakhle, C. Gallou-Kabani, B. Guarmit, A. Morin, N. Prevarskaya, N.B. Delongchamps, F. Cabon, Thrombospondin-1 Triggers Cell Migration and Development of Advanced Prostate Tumors, Cancer research, 71 (2011) 7649-7658.
[80] S.R. Amend, O. Uluckan, M. Hurchla, D. Leib, D.V. Novack, M. Silva, W. Frazier, K.N. Weilbaecher, Thrombospondin-1 Regulates Bone Homeostasis Through Effects on Bone Matrix Integrity and Nitric Oxide Signaling in Osteoclasts, Journal of Bone and Mineral Research, 30 (2015) 106-115.
[81] K.B. DuBose, M. Zayzafoon, J.E. Murphy-Ullrich, Thrombospondin-1 inhibits osteogenic differentiation of human mesenchymal stem cells through latent TGF-beta activation, Biochemical and biophysical research communications, 422 (2012) 488-493.
[82] V. Varma, A. Yao-Borengasser, A.M. Bodles, N. Rasouli, B. Phanavanh, G.T. Nolen, E.M. Kern, R. Nagarajan, H.J. Spencer, M.J. Lee, S.K. Fried, R.E. McGehee, C.A. Peterson, P.A. Kern, Thrombospondin-1 is an adipokine associated with obesity, adipose inflammation, and insulin resistance, Diabetes, 57 (2008) 432-439.
[83] S. Li, S. Ibaragi, G.F. Hu, Angiogenin as a molecular target for the treatment of prostate cancer, Curr Cancer Ther Rev, 7 (2011) 83-90.
[84] Y. Morita, H. Matsuyama, A. Serizawa, T. Takeya, H. Kawakami, Identification of angiogenin as the osteoclastic bone resorption-inhibitory factor in bovine milk, Bone, 42 (2008) 380-387.
[85] J.L. Hao, P.J. Cozzi, A. Khatri, C.A. Power, Y. Li, CD147/EMMPRIN and CD44 are Potential Therapeutic Targets for Metastatic Prostate Cancer, Curr Cancer Drug Tar, 10 (2010) 287-306.
[86] N. Rucci, D. Millimaggi, M. Mari, A. Del Fattore, M. Bologna, A. Teti, A. Angelucci, V. Dolo, Receptor Activator of NF-kappa B Ligand Enhances Breast Cancer-Induced Osteolytic

Lesions through Upregulation of Extracellular Matrix Metalloproteinase Inducer/CD147, Cancer research, 70 (2010) 6150-6160.
[87] Q. Lu, G. Lv, A. Kim, J.M. Ha, S. Kim, Expression and clinical significance of extracellular matrix metalloproteinase inducer, EMMPRIN/CD147, in human osteosarcoma, Oncol Lett, 5 (2013) 201-207.
[88] J. Hao, H. Chen, M.C. Madigan, P.J. Cozzi, J. Beretov, W. Xiao, W.J. Delprado, P.J. Russell, Y. Li, Co-expression of CD147 (EMMPRIN), CD44v3-10, MDR1 and monocarboxylate transporters is associated with prostate cancer drug resistance and progression, British journal of cancer, 103 (2010) 1008-1018.
